# Dlk1 maintains adult mice long-term HSCs by activating Notch signaling to restrict mitochondrial metabolism

**DOI:** 10.1186/s40164-022-00369-9

**Published:** 2023-01-18

**Authors:** Deyu Huang, Yingli Han, Tian Tang, Lin Yang, Penglei Jiang, Wenchang Qian, Zhaoru Zhang, Xinyue Qian, Xin Zeng, Pengxu Qian

**Affiliations:** 1 Center of Stem Cell and Regenerative Medicine, and Bone Marrow Transplantation Center of the First Affiliated Hospital, Zhejiang University School of Medicine, Hangzhou, 310058 China; 2 Liangzhu Laboratory, Zhejiang University Medical Center, 1369 West Wenyi Road, Hangzhou, China; 3 Institute of Hematology, Zhejiang University and Zhejiang Engineering Laboratory for Stem Cell and Immunotherapy, Hangzhou, 310058 China; 4 Dr. Li Dak Sum & Yip Yio Chin Center for Stem Cell and Regenerative Medicine, Zhejiang University, Hangzhou, 310012 Zhejiang People’s Republic of China; 5 Department of Biomedical Sciences, City University of Hong Kong, Kowloon, Hong Kong SAR China

**Keywords:** Dlk1, HSCs, Mitochondrial metabolism, Notch, HSC transplantation

## Abstract

**Background:**

Adult hematopoietic stem cells (HSCs) homeostasis is critically important in maintaining lifelong hematopoiesis. However, how adult HSCs orchestrate its homeostasis remains not fully understood. Imprinted gene Dlk1 has been shown to play critical role in mouse embryonic hematopoiesis and in regulation of stem cells, but its physiological roles in adult HSCs are unknown.

**Methods:**

We performed gene expression analysis of Dlk1, and constructed conditional Dlk1 knockout (KO) mice by crossing Mx1 cre mice with Dlk^flox/flox^ mice. Western blot and quantitative PCR were used to detect Dlk1 KO efficiency. Flow cytometry was performed to investigate the effects of Dlk1 KO on HSCs, progenitors and linage cells in primary mice. Competitive HSCs transplantation and secondary transplantation was used to examine the effects of Dlk1 KO on long-term hematopoietic repopulation potential of HSCs. RNA-Seq and cell metabolism assays was used to determine the underlying mechanisms.

**Results:**

Dlk1 was highly expressed in adult mice long-term HSCs (LT-HSCs) relative to progenitors and mature lineage cells. Dlk1 KO in adult mice HSCs drove HSCs enter active cell cycle, and expanded phenotypical LT-HSCs, but undermined its long-term hematopoietic repopulation potential. Dlk1 KO resulted in an increase in HSCs’ metabolic activity, including glucose uptake, ribosomal translation, mitochondrial metabolism and ROS production, which impaired HSCs function. Further, Dlk1 KO in adult mice HSCs attenuated Notch signaling, and re-activation of Notch signaling under Dlk1 KO decreased the mitochondrial activity and ROS production, and rescued the changes in frequency and absolute number of HSCs. Scavenging ROS by antioxidant N-acetylcysteine could inhibit mitochondrial metabolic activity, and rescue the changes in HSCs caused by Dlk1 KO.

**Conclusion:**

Our study showed that Dlk1 played an essential role in maintaining HSC homeostasis, which is realized by governing cell cycle and restricting mitochondrial metabolic activity.

**Supplementary Information:**

The online version contains supplementary material available at 10.1186/s40164-022-00369-9.

## Background

Hematopoietic stem cells are the seed of the whole hematopoietic system owing to the HSCs homeostasis that relies on balance between self-renewal and differentiation. This balance could directly change HSCs states and affect the lifelong hematopoiesis. HSCs quiescence is a key part of HSCs homeostasis and can influence the self-renewal, and was largely decided by HSC cell division rate. Generally, long-term HSCs with the strongest self-renewal ability cycle very rarely and mainly dwell on G0 phase of cell cycle. Short-term-HSCs (ST-HSCs) and multipotent progenitor (MPP) cycle rapidly to continuously supplement mature cells, most of which are short-lived [1, 2]. However, how adult HSCs orchestrate its homeostasis remains not fully understood. Previous studies have proposed that mitochondrial metabolic properties were of great significance in determining the HSCs quiescence [2–4]. Mitochondria in adult LT-HSCs are relatively inactive compared with MPP and mature lineage cells [5, 6], which maintains low mitochondrial activity and low ROS level. Besides, adult HSCs preferentially resided in bone marrow niches that were hypoxic with low ROS [5]. Thus, low mitochondrial activity and low ROS levels were closely relevant to adult HSCs functionality. But how adult HSCs restrict mitochondrial activity were not completely clear.

Dlk1 was a paternal-expressed imprinted gene located in Dlk1-Gtl2 gene cluster of mice and was known as an inhibitor of adipogenesis [7]. Dlk1 has also been considered as a non-canonical Notch ligand, which could regulate cell proliferation and differentiation, tissue regeneration in Notch-dependent and independent manners [8]. Dlk1 knockout mice exhibited nutritional defects, smaller finger, smaller spleen size and abnormal B cell development [9, 10]. Moreover, Dlk1 was documented as an important regulator of cell metabolism. Dlk1 suppressed GLUT4-mediated glucose uptake and directly and negatively regulated overall glucose homeostasis upon obesity [11]. Increased expression level of Dlk1 was closely related to insulin resistance [12]. Dlk1 also promoted fatty acid oxidation to enhance cellular metabolic level in diabetes mouse model and maternal pregnancy period [13, 14]. Dlk1 could impact mitochondrial activities, including mitochondrial membrane potential, ROS production and mitochondria biogenesis [2, 15].

Dlk1 was reported to be required for fetal hematopoiesis. In the early development stage (E10.5 when the first HSCs with long-term hematopoiesis potential appeared) of fetal liver, there was a small group of cells expressing high level of Dlk1, and they possessed the most robust hematopoietic colony formation ability [16]. Dlk1 expressed in aorta-gonad-mesonephros region (AGM) can negatively regulate hematopoietic stem and progenitor cell activity in vivo, and also in in vitro co-culture system (AGM-derived stromal cells and HSCs), Dlk1 expressed in stromal cells with varying levels of Dlk1 could limit HSCs activity [17]. Our previous study and others have demonstrated the crucial roles of Dlk1-Gtl2 gene cluster in embryonic and adult stem cells [2, 18, 19]. Dlk1 was also involved in hematologic malignancies. It was reported that Dlk1 was highly expressed in majority of CD34^+^ cells isolated from myelodysplastic syndromes patients and acute myeloid leukemia samples [20, 21]. It also had a high mRNA level in erythroleukemia and megakaryocytic leukemia cell lines. In HL-60 cell line, transmembrane domain of Dlk1 could impede cell proliferation while intracellular region could block cell differentiation [20]. Exogenous expression of Dlk1 in K562 was also able to control cell division and differentiation [9, 22].

In this study, we observed that Dlk1 was highly expressed in adult mice long-term HSCs, and gradually decrease its expression when differentiated into progenitors and lineage cells. This hinted us that Dlk1 might have a unique role in adult mice long-term HSCs. To explore the function of Dlk1, we specifically knockout Dlk1 in HSCs of mice by Mx1-Cre/loxP system. We found that Dlk1 knockout could result in a significant increase in frequency and absolute number of phenotypical LT-HSCs. However, Dlk1-deficient LT-HSCs exhibited a remarkable decrease in the long-term hematopoietic repopulation potential compared with the controls. Besides, we showed that Dlk1 knockout in adult mice HSCs could increase the cell cycle entry into S-G2-M state, and also increase the ribosomal translation, mitochondrial metabolism and ROS production. Mechanically, Dlk1 knockout could attenuate the Notch signaling, which impaired the function of HSCs and increase the mitochondrial activity and activate cell cycle. Thus, this study revealed a new role for Dlk1 in maintaining adult mice HSCs homeostasis through governing the cell cycle and restricting mitochondrial metabolism.

## Materials and methods

### Mice

Dlk1 conditional KO mice was generated by Jennifer Schmidt, University of Illinois at Chicago, and we purchased from Jackson lab. ptprc mutant mice and Mx1-cre mice were purchased from Shanghai Model organisms. All mice were housed under specific pathogen-free conditions in the Laboratory Animal Center of Zhejiang University (ZJU). The genotype identification of mice offspring was performed 3–4 weeks after birth. The mice offspring were intraperitoneally injected every other day with 2 mg/kg b.w. poly I:C for 7 times after genotype identification. The control mice were treated with the same conditions with the Dlk1 knockout mice. The flow cytometric analysis was carried out at least 1 month after injection of poly I:C.

### Cell culture

The bone marrow cells derived from mice were culture in vitro in 96 well plate in StemSpan™ SFEM II (Stem Cell Technologies, #09655) with mouse five growth factors (SCF, TPO, FLT-3L, IL3 and IL-6) (novoprotein) for 7 days. At the second day, a final concentration of 10 mM of N-acetylcysteine (NAC, sigma, C7352-25G) or 50 ng/mL JAG1 (Abcam, ab109346) was added and at the fifth day, the medium in 96 well plate was replaced with fresh medium containing 10 mM NAC or 50 ng/mL JAG1. At the seventh day, the cells were harvested, counted. The HSCs gated from LSK, CD150^+^, CD48^−^ was analyzed by flow cytometry for frequency and mitochondrial parameters.

### Flow cytometry and LSK sorting

Mice mononuclear cells were collected from bone marrow, spleen and peripheral blood that were further lysed to remove the red blood cells. For mouse HSCs identification, mononuclear cells were stained with antibodies against Sca-1 (D7, biolegend, #108114), c-Kit (2B8, eBioscience, #17-1171-83), CD34 (RAM34, eBioscience, #11–0341-85), Flk2 (A2F10, BD, #562898), CD48 (HM48-1, eBioscience, #48-0481-82), CD150 (TC15-12F12.2, BioLegend, #115904), along with lineage cocktail including CD3e (145-2C11, eBioscience, #17-0031-83), CD4 (RM4-5, eBioscience, #15-0042-83), CD8a (53–6.7, eBioscience, #15-0081-83), Mac-1 (M1/70, eBioscience, #15-0112-83), Gr1 (RB6-8C5, eBioscience, #15–5931-83), CD45R (B220, RA3-6B2, eBioscience, #15–0452-83), IgM (Il-41, eBioscience, #15-5790-82), Ter119 (TER-119, eBioscience, #15-5921-83), CD127 (A7R34, eBioscience, #48-1271-82), CD16/CD32 (93, eBioscience, #25-0161-82), CD45.2 (eFluor780, eBioscience, #47-0454-82), CD45.1 (A20, eBioscience, #15-0453-82). Antibodies against CD3, CD4, CD8, B220, IgM, Ter119, CD71, Mac1 and Gr1 were utilized for lineages clarifying. As for donor engraftment analysis, mononuclear cells isolated from peripheral blood were stained with antibodies against CD45.1, CD45.2, Mac1, Gr1, CD3, B220. To identify progenitors, cells were stained with antibodies against Sca-1, c-Kit, CD34, CD16/32, CD127 and lineage cocktail. Antibodies against lineage cocktail, Sca-1 and c-Kit were used to stain the LSK for sorting that is conducted on MoFlo (Dako).

### Metabolism detection assays

*Glucose uptake detection assay:* mononuclear cells were stained with HSCs markers and then marked with 2-NBDG (Life technologies, N13195) at 37 °C for 1 h.

*Translation efficiency:* cells staining with HSCs markers were incubated with OP-puro from the Click-iT™ Plus OPP Alexa Fluor™ 488 Protein Synthesis Assay Kit (ThermoFisher Scientific, C10456) at 37 °C for 1 h.

*Mitochondrial function analysis:* after staining with HSCs markers, cells were incubated with multiple fluorescence probes. Cells were incubated with 20 nM Mito Tracker Green (Beyotime, C1048) at 37 °C for 30 min to measure mitochondrial mass; 50 nM DilC-5 (Invitrogen) and 50 nM TMRE (Beyotime, C2001S) were utilized at 37 °C for 30 min for MMP assessment. For ROS analysis, cells were stained with 2 uM DCFH-DA (Invitrogen, C2938) and 50 nM mitoSOX red (Life technologies, M36008) at 37 °C for 30 min.

*mito-DNA copy number:* Lineage negative cells were sorted with mouse lineage cell deletion kit (Miltenyibiotec, #130-090-858) according to its manufacturer’s instructions. Genomic DNA of sorted cells were extracted with cell/tissue DNA isolation kit (Vazyme, DC102) and qPCR assay (Taq Pro Universal SYBR qPCR Master Mix, Q712) was performed to detect relative quantity of mitochondrial gene, ND4 and Cox2.

*ATP detection assay:* lineage negative cells were collected with above method and the ATP level was analysis with ATP detection kit (Beyotime Biotechnology, S0026).

### Transplantation assay

Ptprc stain mice were bred by baytril water for 1 week before and after transplantation and irradiated with nine lethally irradiated (9 Gy) in twice at the previous day. For ELDA assay, 2 × 10^5^, 7.5 × 10^4^, or 2.5 × 10^4^ donor-derived bone marrow mononuclear cells (BMMCs) from WT or Dlk1 KO mice (CD45.2), together with 2 × 10^5^ rescue cells (CD45.1) were transplanted intravenously into ptprc recipient mice. For secondary transplantation, 1 × 10^6^ BMMCs derived from primary transplanted mice were mouse-to-mouse transplanted into secondary recipient mice. For competitive transplantation assay using donors under long-term Dlk1 knockout, the procedures were nearly same as ELDA assay, but it only transplanted 2 × 10^5^ donor cells and 2 × 10^5^ rescue cells. In terms of invert transplantation, ptprc stain mice were served as donor while WT and Dlk1 KO mice were considered as recipient, the pretreatment and experimental procedures are the same to the competitive transplantation. Briefly, 2 × 10^5^ BMMCs derived from ptprc mice and 2 × 10^5^ BMMCs isolated from CD45.2 mice were intravenously transplanted into WT and Dlk1 KO mice. For reciprocal transplantation, 500 HSCs from wild-type congenic CD45.1 mouse were transplanted into Mx-1 Cre induced Dlk1 WT or Dlk1 KO CD45.2 recipients. The recipients were intraperitoneally injected with poly I:C to induce the expression of Mx-1 Cre 1 month after birth, and were lethally irradiated at the previous day of transplantation. Overall donor engraftment was confirmed by flow cytometric analysis.

### Cell cycle and apoptosis assays

According to the manufacturer’s instructions of BD Pharmingen™ FITC Mouse Anti-Ki-67 Set (BD Pharmingen™, #556026), 5 million mononuclear cells stained with HSCs markers as described above were fixed and permeated with fixation/permeabilization buffer at 4 °C for 1 h, and then washed with PBS containing 2% fetal bovine serum. After permeabilization, cells were incubated with anti-Ki-67 at 4 °C for 1 h in the dark and then stained with DAPI (Sango, E607303) at room temperature (RT) for 10 min. As for apoptosis assay, cells also conducted with HSCs antibodies stained with Annexin-V (Yeasen, 40304ES60) or 7-AAD (Sango, A606804) at RT for 10–15 min.

### RNA sequencing

Three replications of LSK cells were sorted and total RNA were extracted with TRizol (Takara, 9109). High quality total RNA was reverse transcript to cDNA for library preparation using the SMART-Seq v4 Ultra Low Input RNA Kit for Sequencing (Takara, 634832) and Nextera XT (Illumina, FC-131–1024) according to the manufacturers’ protocols.

### qRT-PCR analysis

Lineage negative cells were isolated from WT and Dlk1 KO mice and total RNA was extracted by TRizol (Takara, 9109) according to its instruction. 200 ng total RNA were used for reverse transcription with HiScript II 1st Strand cDNA Synthesis Kit (Vazyme, R212) and cDNA were used for qPCR.

### Western blot

For measuring protein level of Dlk1 and key components of Notch pathway, lineage negative cells were isolated and cellular protein were extracted with RIPA lysis buffer following the manufacturer’s protocol. Immunoblotting was performed with anti-Dlk1 mouse monoclonal antibody (Santa cruz, sc-376755), Notch 1 mouse monoclonal antibody (Santa cruz, sc-376403), Jagged 1 mouse monoclonal antibody (Santa cruz, sc-390177), Hes 1 mouse monoclonal antibody (Santa cruz, sc-166410) and β-actin mouse monoclonal antibody (Sangon, D191047), HEY1 rabbit pAb (abclonal, A16110), HES5 rabbit pAb (abclonal, A16237), Notch3 rabbit pAb (abclonal, A13522), Notch1 NICD rabbit mAb (abclonal, A19090). The goat anti-mouse or anti-rabbit secondary antibody was purchased from Sangon.

### Statistical analysis

Comparison between two groups was analyzed by unpaired two tailed Mann–Whitney test (non-parametric test). Comparison between multiple groups was analyzed by Kruskal Wallis test. Data were shown as mean ± SD. Statistical significance was defined as p < 0.05. Majority of graphs were generated by GraphPad Prism 8 (GraphPad Software). For CRUs in ELDA assay, data were analyzed by ELDA software. Successful engraftment was defined as that the frequency of CD45.2^+^ CD45.1^−^ population is above 5% of total hematopoietic cells in peripheral blood.

## Results

### Dlk1 was predominantly expressed in LT-HSCs

**Fig. 1:**
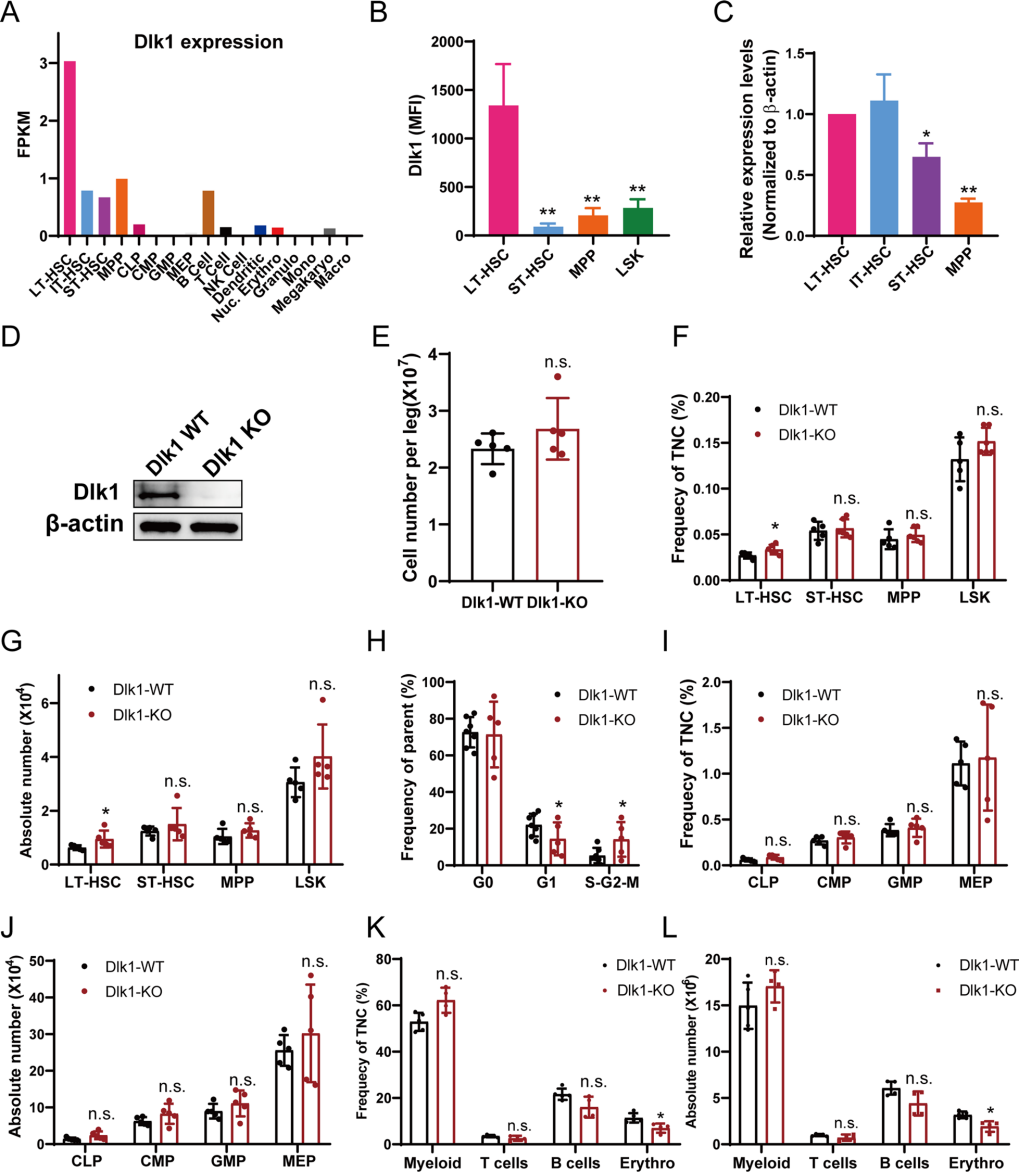
Dlk1 was highly expressed in LT-HSCs and Mx1-Cre-mediated Dlk1 knockout in mice increased number of LT-HSCs. **A**–**C** The expression of Dlk1 was analyzed in 17 mice hematopoietic cell types in a previous study from us, and was validated by flow cytometry (**B**) and qRT-PCR (**C**). **D** Western blot result validating that Dlk1 was almost completely knocked out after poly I:C inducement. The LSK were lysed for protein loading. **E**–**L** The changes in bone marrow mononuclear cells (**E**), frequency and absolute number of HSPC (**F** and **G**), cell cycle state of HSC (**H**), frequency and absolute number of progenitor (**I** and **J**) and frequency and absolute number of linage cells (**K** and **L**) in primary Dlk1 KO and WT mice after poly I:C inducement (n = 5). Data were expressed as mean ± SD; *p < 0.05; **p < 0.01. WT indicated wild-type mice; KO indicated knockout mice; TNC indicated total nuclear cells. n.s. indicated no significance

In our previous study, 17 types of murine hematopoietic cells were sorted by fluorescence-activated cell sorting and used for RNA-seq [2]. This RNA-seq result displayed that Dlk1 was highly expressed in LT-HSCs (Lin^−^, c-Kit^+^, Sca1^+^, CD34^−^, Flk2^−^ and CD49b^low^) at both mRNA and protein level, the latter of which was validated by flow cytometry (Fig. 1A, B). qRT-PCR was also exploited to validate the expression of Dlk1 and showed a similar result (Fig. 1C). Several online hematological databases were also examined to systematically analyze the expression of Dlk1 in diverse blood cells. Bloodspot database exhibited that Dlk1 presented highest expression in HSCs, with relatively moderate level in MPP and low level in progenitors and mature cells (Additional file 1: Fig. S1A). Haemosphere database also showed that Dlk1 has a highest expression in LT-HSCs and moderate expression in ST-HSCs and MPP (Additional file 1: Fig. S1B). Overall, these results hinted that Dlk1 might play a unique role in LT-HSCs.

### Mx1-Cre-mediated Dlk1 knockout in mice increased number of LT-HSCs

The Mx1-cre/floxp platform was used to specifically knockout Dlk1 in adult mice HSCs, in which the last two exons of Dlk1 were flanked by loxP sites (Additional file 1: Fig. S1C) [23]. After poly I:C induction, the protein expression of Dlk1 was examined and showed that Dlk1 was almost completely knocked out (KO) in HSCs (Fig. 1D). Interestingly, we observed a significant increase in frequency and absolute number of LT-HSCs in Dlk1 knockout mice, although total number of bone marrow cells were almost unchanged (Fig. 1E–G, Additional file 2: Fig. S2A). Analysis of cell cycle of HSCs showed that Dlk1 knockout resulted in pronounced higher frequency of S-G2-M stage cells, suggesting that Dlk1 deficiency facilitated the entry of quiescent HSCs into the cell cycle (Fig. 1H). We further examined the committed progenitors and mature lineage cells, and found that Dlk1 deletion did not exhibit obvious alterations in frequency and absolute number of common lymphoid progenitor (CLP), common myeloid progenitors (CMP), granulocyte–macrophage progenitors (GMP), megakaryocyte-erythrocyte progenitors (MEP) (Fig. 1I, J, Additional file 2: Fig. S2B). We further observed that Dlk1 knockout did not result in obvious alterations in frequency and absolute number of myeloid, T cells, except that B cells were slightly decreased although with no significance, and erythrocyte (erythro as abbreviation in figures) showed a significant reduction (Fig. 1K, L, Additional file 2: Fig. S2C). Together, these results implied that Dlk1 KO could increase the number of phenotypical LT-HSCs,.

### Dlk1 deletion did not render malignant hematopoiesis

To address the concern that whether HSC expansion caused by Dlk1 deletion has any detrimental effects on normal hematopoiesis, we examined the HSCs in the Dlk1 knockout mice 8 months after injection of poly I:C. We showed that the spleen size, spleen total cell number, the frequency and absolute number of HSPCs, progenitors in the spleen were not evidently changed (Additional file 3: Fig. S3A–I). We also examined the hemograms and found that a modest decrease in number of white blood cells (WBC), number and frequency of lymphocytes, frequency of HCT, MCV and PCT, as well as an increase in frequency of granulocytes (Additional file 3: Fig. S3J), which were not the classic leukemia symptoms. Thus, we reckoned that Dlk1 knockout in hemopoietic system would not render malignant hematopoiesis.

### Dlk1 knockout undermined the repopulating capacity of LT-HSCs

**Fig. 2:**
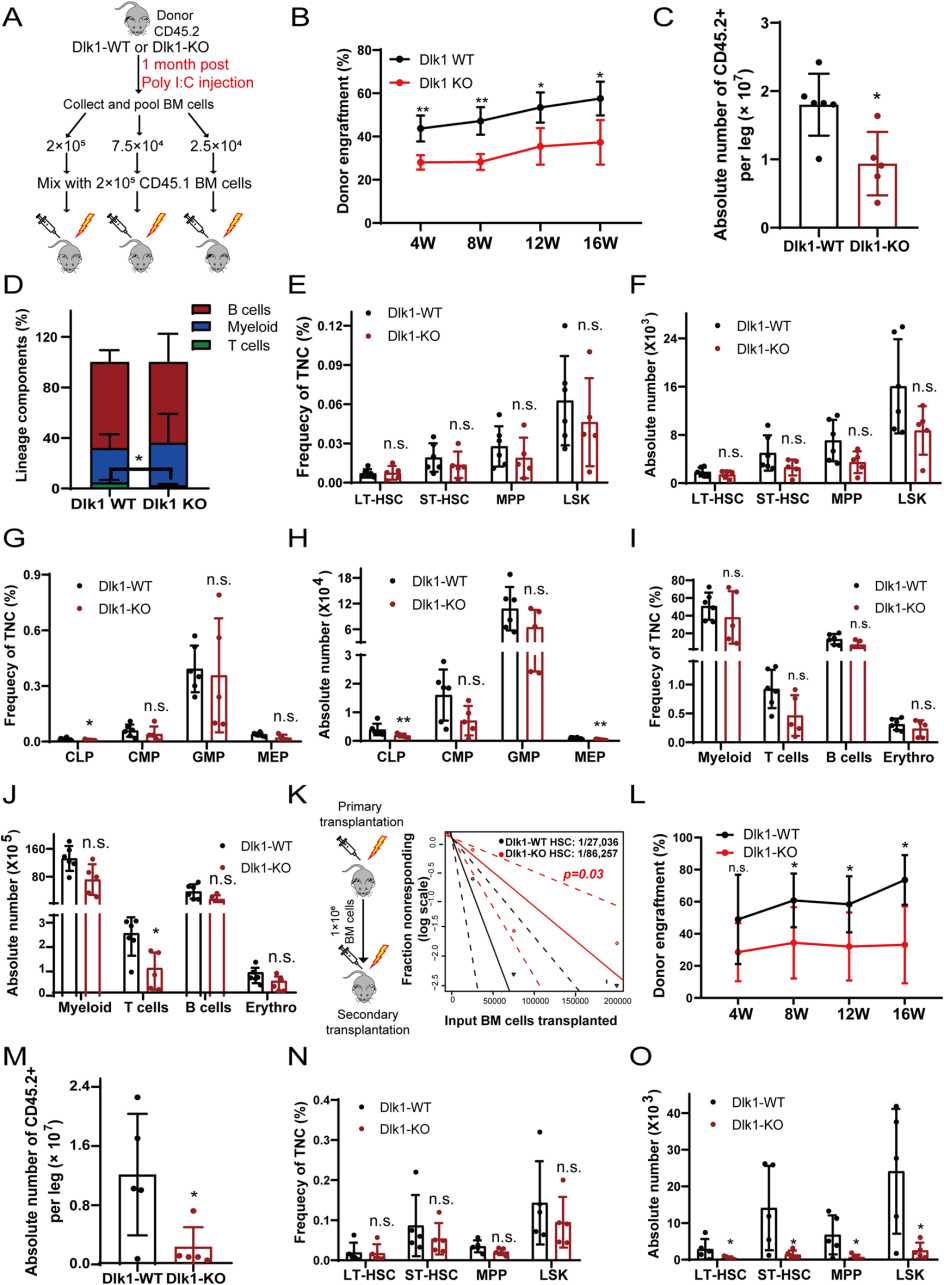
Dlk1 knockout in adult mice HSCs dampened long-term hematopoietic repopulation potential. **A** Schematic diagram of the ELDA assay. 2 × 10^5^, 7.5 × 10^4^ or 2.5 × 10^4^ donor-derived bone marrow mononuclear cell from WT or Dlk1 KO mice (CD45.2) 1 month post poly I:C injection were transplanted with 2 × 10^5^ rescue cells (CD45.1) into irradiated recipients. **B** Peripheral blood (PB) of 2 × 10^5^ group was analyzed for percent donor repopulation at the indicated number of weeks after transplants (n = 6). **C** The absolute number of donor-derived BM cells was analyzed in Dlk1 WT and KO 1st recipients at the end of ELDA assay (n = 6). **D** PB of the mice at the end of ELDA assay was analyzed for percent mature donor-derived lineage cells (n = 6). **E**–**J** Frequency in total nuclear cells (TNCs) and absolute numbers of HSPCs (**E** and **F**), progenitors (**G** and **H**) and lineage cells (**I** and **J**) in Dlk1 WT and KO 1st recipient mice (n = 6). **K** At 16 weeks posttransplant, 1 × 10^6^ BMMCs isolated from 1^st^ recipients were transplanted into 2nd recipients. CRU frequency was determined using ELDA (Extreme Limiting Dilution Analysis). **L** PB of 7.5 × 10^4^ group in 2nd recipients were analyzed for percent donor repopulation at the indicated number of weeks post-transplantation (n = 6). **M** Absolute number of donor-derived BM cells in Dlk1 WT and KO 2nd recipients (n = 6). **N**–**O** Frequency in TNCs and absolute numbers of HSPCs in 2nd recipients were analyzed (n = 6). Data were expressed as mean ± SD; *p < 0.05; **p < 0.01; ***p < 0.001

To identify the functional roles of Dlk1 in hematopoietic reconstitution ability of HSCs, we performed extreme limiting dilution analysis (ELDA), as shown in Fig. 2A. Interestingly, we found that the repopulating potential of Dlk1 knockout HSCs was notably undermined, as manifested by reduction of the overall engraftment and the absolute number of donor cells (CD45.2) from Dlk1 knockout mice when compared with their littermates (Fig. 2B, C). We also observed a moderate decrease in T cells of Dlk1 knockout recipients after 16 weeks of transplantation, but with no obvious changes in B cells and myeloid (Fig. 2D). A more thorough analysis of the bone marrow of recipient mice showed that the absolute number and frequency of various donor cells, including LSK (Lin^−^, c-Kit^+^, Sca1^+^), LT-HSC (LSK, CD34^−^ and Flk2^−^), ST-HSC (LSK, CD34^+^ and Flk2^−^), MPP (LSK, CD34^+^ and Flk2^+^), CLP, CMP, GMP, MEP and mature lineages (myeloid, T cells, B cells and erythrocyte) were reduced with different degrees (Fig. 2E–J, Additional file 4: Fig. S4A–C). To further verify long-term outcomes of Dlk1 knockout in hemopoietic system, we conducted secondary transplantation (Fig. 2K). We also observed a significant decrease in chimerism rate of Dlk1 knockout group (Fig. 2L). ELDA software was utilized to assess the absolute number of functional HSCs in total transplanted cells [24]. We showed that competitive repopulating units (CRUs) in Dlk1 wild type group was 1/27036 while CRUs in Dlk1 knockout group was 1/86257, which was 3.2-fold lower than wild-type group (Fig. 2K). Also, analysis of the bone marrow of the secondary recipient mice revealed that absolute number of bone marrow cells and LT-HSC, ST-HSC, MPP, LSK from recipient mice were significantly reduced (Fig. 2M, O), although the frequency of LT-HSC, ST-HSC, MPP, LSK showed a descending trend with no significance (Fig. 2N, Additional file 4: Fig. S4D).

**Fig. 3:**
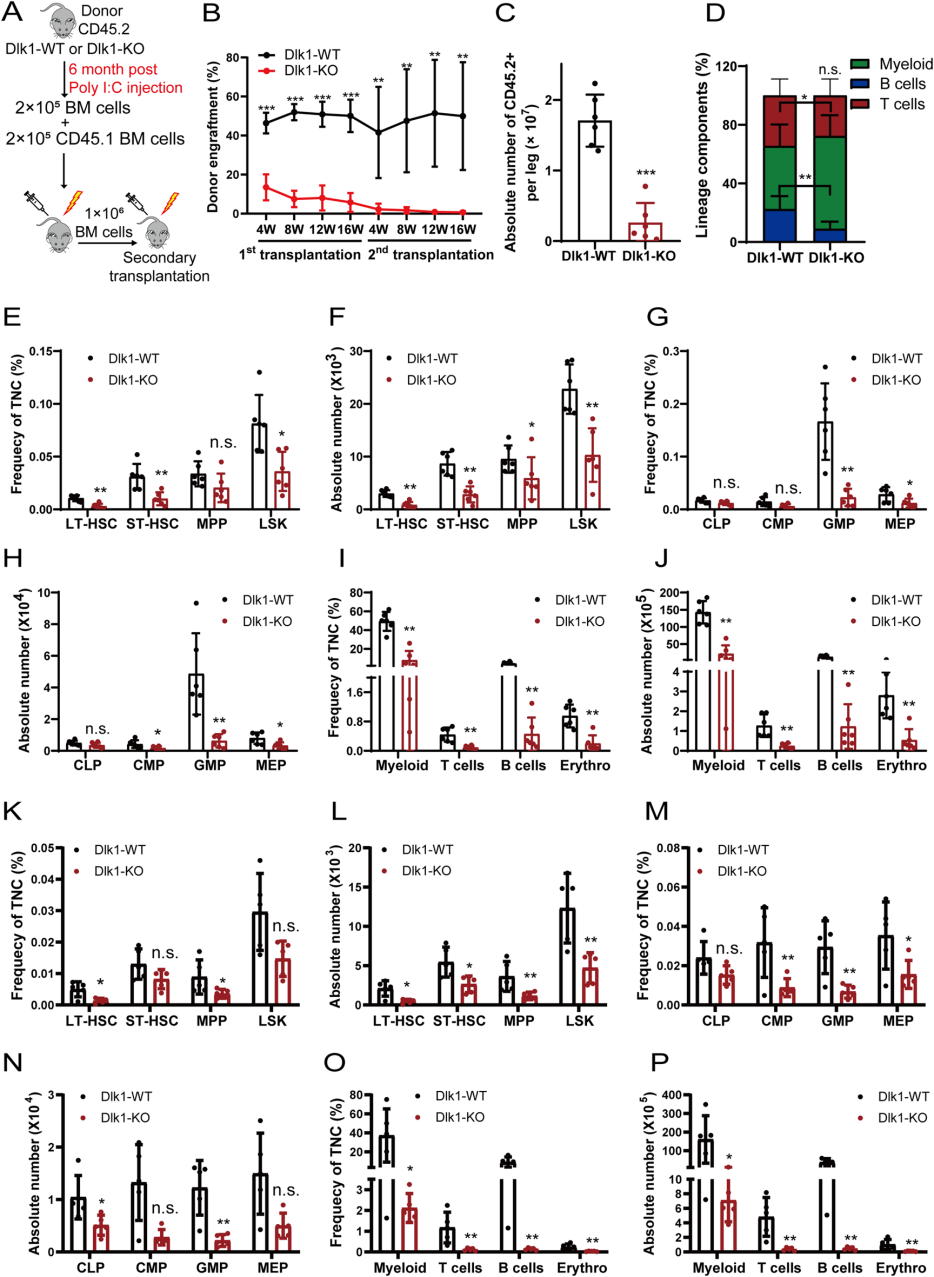
Dlk1 knockout in adult mice HSCs dampened long-term hematopoietic repopulation potential. **A** 2 × 10^5^ donor-derived BMMCs from Dlk1 WT and KO mice (CD45.2) 6 months post poly I:C injection were transplanted with 2 × 10^5^ rescue cells (CD45.1) into irradiated recipients. At 16 weeks posttransplant, 1 × 10^6^ BMMCs isolated from 1st recipients were transplanted into 2nd recipients. **B** PB was analyzed for percent donor repopulation at the indicated number of weeks after transplants in Dlk1 WT and KO 1st and 2nd recipients (n = 6). **C** The absolute number of donor-derived BM cells in Dlk1 WT and KO 1st recipients were analyzed (n = 6). **D** PB of 1st recipients were analyzed for percent mature donor-derived lineage cells at 12-week posttransplant (n = 6). **E**–**J** Frequency in TNCs and absolute numbers of HSPCs (**E** and **F**), progenitors (**G** and **H**) and lineage cells (**I** and **J**) in Dlk1 WT and KO 1st recipient mice (n = 6). **K**–**P** Frequency in TNCs and absolute numbers of HSPCs (**K** and **L**), progenitors (**M** and **N**) and lineage cells (**O** and **P**) in Dlk1 WT and KO 2nd recipients (n = 6). Data were expressed as mean ± SD; *p < 0.05; **p < 0.01; ***p < 0.001

We further characterized the functional roles of Dlk1 in HSCs with competitive repopulation assay, which the donor mice were sustained for a longer time of 6 months after poly I:C inducement (Fig. 3A). In line with the results of the ELDA assay, we observed that Dlk1 knockout in HSCs dramatically decreased its hematopoietic repopulation potential after series transplantation (Fig. 3B) and significantly decreased the absolute number of donor cells with a greater degree compared with that of ELDA assay (Figs. 2C, 3C). We also observed an obvious myeloid lineage bias and inhibition of B cells and T cells in first transplantation recipients of Dlk1 knockout group after 12 weeks of transplantation (Fig. 3D). The frequency and absolute number of almost all kinds of donor-derived cells in first transplantation recipients were decreased to varying degrees (Fig. 3E–J, Additional file 5: Fig. S5A–C). The results of secondary transplantation were similar to that of first transplantation (Fig. 3K–P). Notably, the mature lineages of T cells, B cells and erythrocyte, as well as the chimerism rate were approaching to zero after 16 weeks of secondary transplantation (Fig. 3B, O, P, Additional file 6: Fig. S6A–C). Taken together, these data indicated that Dlk1 knockout in HSCs would impair the long-term hematopoietic repopulation potential of HSCs.

### Phenotypes of Dlk1 KO did not rely on hematopoietic microenvironment

Given that the full-length Dlk1 could be cleaved to produce a secreted protein with biological functions [25]. It has been reported that the secreted form of Dlk1 could interact with its ligands to regulate cellular physiological activities in a paracrine mode [26]. Besides, bone marrow stromal cells were able to produce Dlk1 [27], which may have an impact on functions of HSCs in an extrinsic way. So, to distinguish whether Dlk1 knockout-mediated effects were due to extrinsic influence of soluble form of Dlk1 in bone marrow microenvironment or intrinsic changes of HSCs, we conducted a reciprocal transplantation assay, in which CD45.1 donor cells were transplanted into Dlk1 knockout or wild type recipients (CD45.2). We showed that Dlk1 knockout in bone marrow microenvironment did not notably change the hematopoietic repopulation potential of CD45.1 donor cells (Additional file 7: Fig. S7A–D). After 16 weeks of transplantation, the frequency and absolute numbers of LT-HSCs, ST-HSCs, IT-HSCs and MPP have no discernible differences between Dlk1 wild type and knockout group (Additional file 7: Fig. S7E, F), indicating that an intrinsic change in the Dlk1 knockout HSCs was the primary cause of the phenotypes.

### Dlk1 deletion contributed to enriched gene signature in mitochondrial activities

**Fig. 4:**
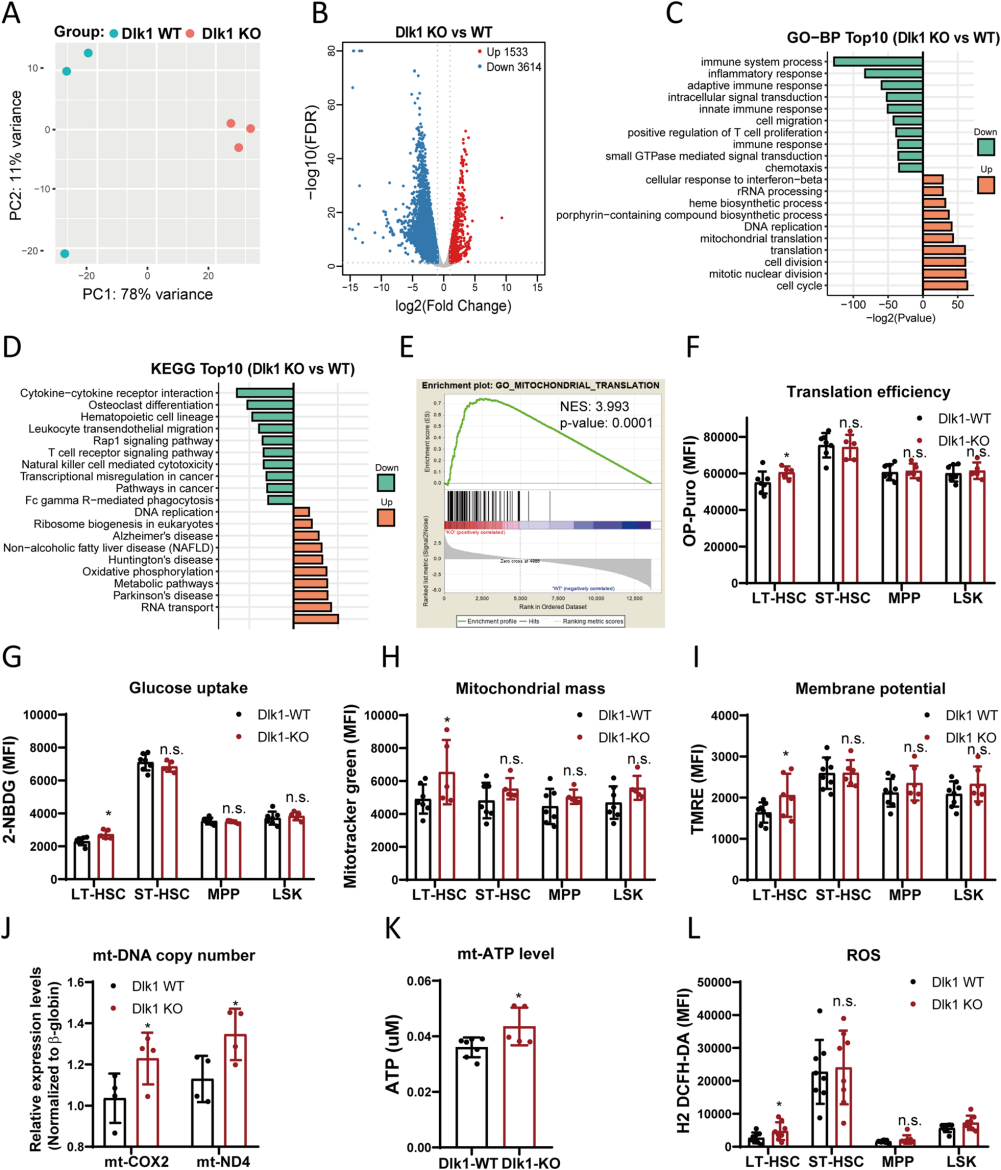
Dlk1 knockout in adult mice HSCs led to hyperactivated mitochondrial metabolism. **A**, **B** PCA analysis and volcano plot analysis of the RNA-Seq data. **C**, **D** Gene ontology terms and KEGG pathways. **E** GSEA analysis of the mitochondrial activity. **F**–**N** Cellular translation and mitochondrial activities detection (n = 4 or 6). Data were expressed as mean ± SD; *p < 0.05; **p < 0.01

To identify the mechanism by which Dlk1 knockout expanded the phenotypical long-term HSCs but impaired its repopulation potential, we sorted LSK cells from Dlk1 WT or KO mice and conducted RNA-sequencing (Additional file 8: Fig. S8A). Unbiased principal component analysis indicated that the expression pattern of Dlk1 wild type or knockout group was diacritical (Fig. 4A). The volcano map revealed that Dlk1 knockout in LSK led to 5147 differentially expressed genes (DEGs), in which 1533 genes were upregulated and 3614 genes were downregulated (Fig. 4B). Gene Ontology (GO) analysis revealed that most of the upregulated terms enriched in cell cycle, mitotic nuclear division, translation, ribosome biogenesis, rRNA processing and mitochondrial translation (Fig. 4C). KEGG analysis also showed that upregulated genes enriched in pathways related to metabolism, oxidation phosphorylation and ribosome (Fig. 4D). GSEA analysis showed that in Dlk1 knockout group, gene signatures related to mitochondrial function and metabolism were upregulated, including mitochondrial gene expression, mitochondrial translation, mitochondrial respiratory chain complex assembly, mitochondrial electron transport, ATP synthesis and oxidation phosphorylation (Fig. 4E, Additional file 8: Fig. S8C). Collectively, these data imply that Dlk1 knockout in adult mice HSCs could activate the cell cycle and enhanced the cellular translation and mitochondrial activities.

### Dlk1 knockout led to enhanced cellular and mitochondrial metabolism

To verify the increased mitochondrial metabolism and ribosomal translation caused by Dlk1 knockout in HSCs, we used multiple fluorescence probes to experimentally measure cellular metabolism and mitochondrial function. We analyzed protein synthesis in Dlk1 knockout HSPCs using an O-propargyl-puromycin (OPP) incorporation assay. In line with the RNA-Seq results, we observed that OPP incorporation was significantly increased in only Dlk1 knockout LT-HSCs, but not in ST-HSCs, MPP and LSK (Fig. 4F). Ribosome function and translation efficiency are important for HSCs homeostasis. In regards to the steady state of adult HSCs, protein synthesis level is low compared with committed cells and is under strict regulation [28]. Dysregulation in ribosome assembly and translation could impair the function of HSCs and even cause hematopoietic disorders, including Diamond-Blackfan anemia (DBA) and MDS [29–31]. Increased protein synthesis tends to require a higher energy demand. We measured the glucose uptake by using 2-NBDG in Dlk1 knockout and wild type HSPCs. We showed that Dlk1 knockout led to significantly increased glucose uptake in LT-HSCs (Fig. 4G). We further examined the mitochondrial mass, mitochondrial DNA copy number and mitochondrial membrane potential (MMP), and showed that these mitochondrial parameters were all significantly increased in Dlk1 knockout LT-HSCs (Fig. 4H–J). The increased mitochondrial mass and MMP represent a higher mitochondrial biogenesis and mitochondrial metabolic activity, and in consistent with this, ATP content also revealed an obvious increase after Dlk1 knockout (Fig. 4K). We speculated that the increased mitochondrial metabolic activity after Dlk1 knockout might produce excessive ROS, which could harm HSCs. Therefore, we examined the ROS level and showed that the overall ROS was significantly increased in LT-HSCs and LSK (Fig. 4L). HSCs, particularly the LT-HSCs have been demonstrated to primarily keep a quiescence state in peacetime, which is maintained by low mitochondrial activity and very low ROS level [32]. A previous study also found that HSCs with lowest mitochondrial activity exhibit greatest hematopoietic potency and stem cell potential [33]. Accordingly, our results indicated that Dlk1 knockout could increase the ribosomal translation and mitochondrial activity in LT-HSCs.

**Fig. 5:**
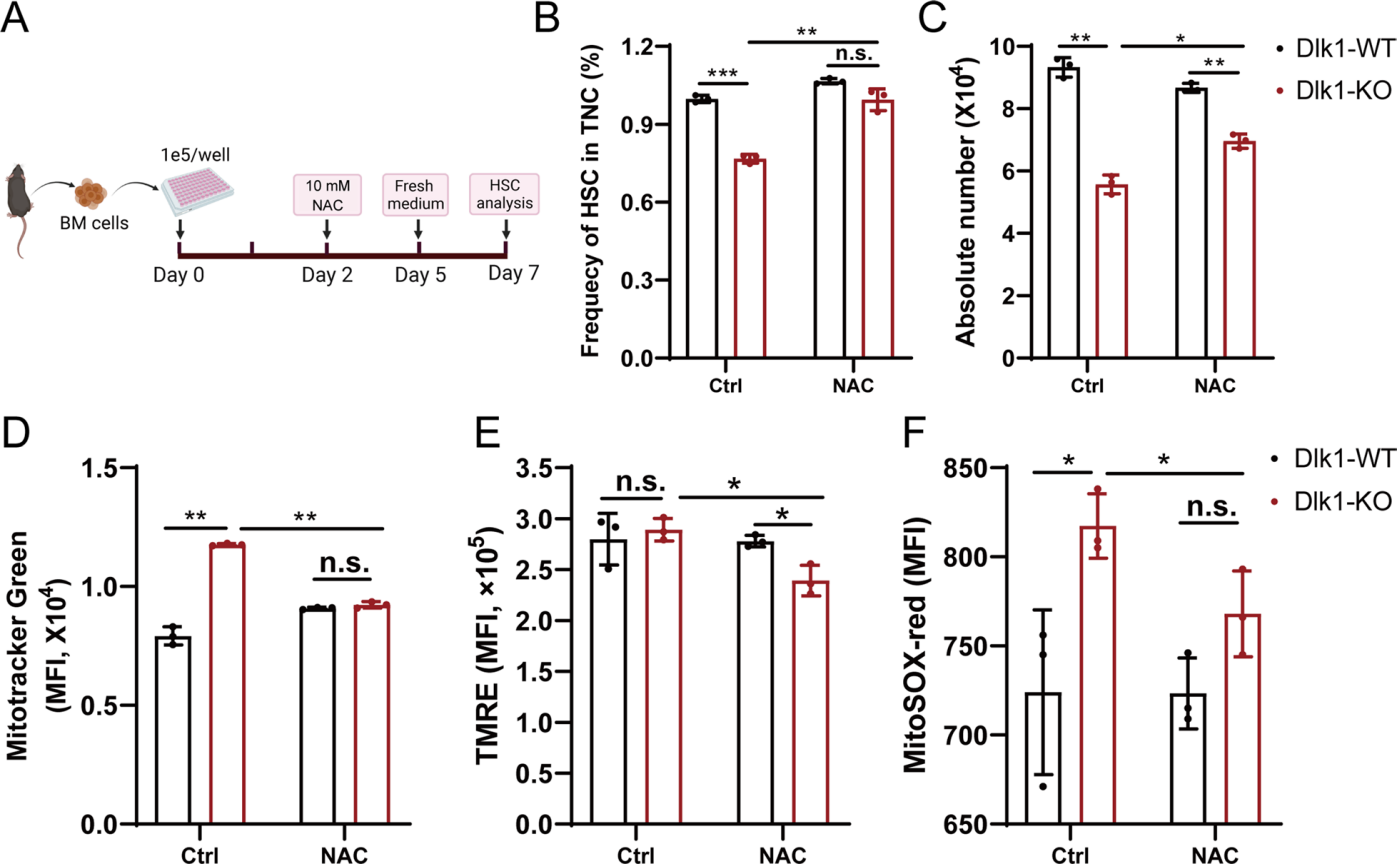
N-acetyl-l-cysteine (NAC) recused the defective phenotypes of HSCs caused by Dlk1 knockout. **A** Schematic diagram of NAC incubation with bone marrow cells during in vitro culture. The SLAM HSCs (LSK, CD150^+^ and CD48^−^) were gated for following analysis. **B**–**C** Frequency in TNC and the absolute number of SLAM HSCs per legs. **D** Analysis of mitochondrial mass by Mitotracker green in HSCs (n = 3). **E** Analysis of mitochondrial membrane potential by TMRE in HSCs (n = 3). **F** Analysis of mitochondrial superoxide by MitoSOX Red in HSCs (n = 3). Data were expressed as mean ± SD; *p < 0.05; **p < 0.01

To test whether the enhanced mitochondrial metabolism was the direct cause of Dlk1 knockout-induced phenotype in HSCs, we used an ROS scavenger, N-acetyl-l-cysteine (NAC) to rescue the phenotypes of HSCs in the context of Dlk1 knockout (Fig. 5A). As shown in Fig. 5, it was revealed that Dlk1 knockout significantly decreased the frequency and absolute number of HSCs, and increased the mitochondrial mass, MMP and superoxide level, which however, could be recovered to a large extent by NAC (Fig. 5B–F). Thus, these results indicated that Dlk1 knockout could expand phenotypic HSCs, which is possibly due to activation of cell cycle and enhanced mitochondrial metabolic activities in LT-HSCs, and consequently led to accumulation of ROS and damage of long-term repopulation potential of LT-HSCs.

### Dlk1 repressed HSCs mitochondrial metabolism through activation of Notch signaling

**Fig. 6:**
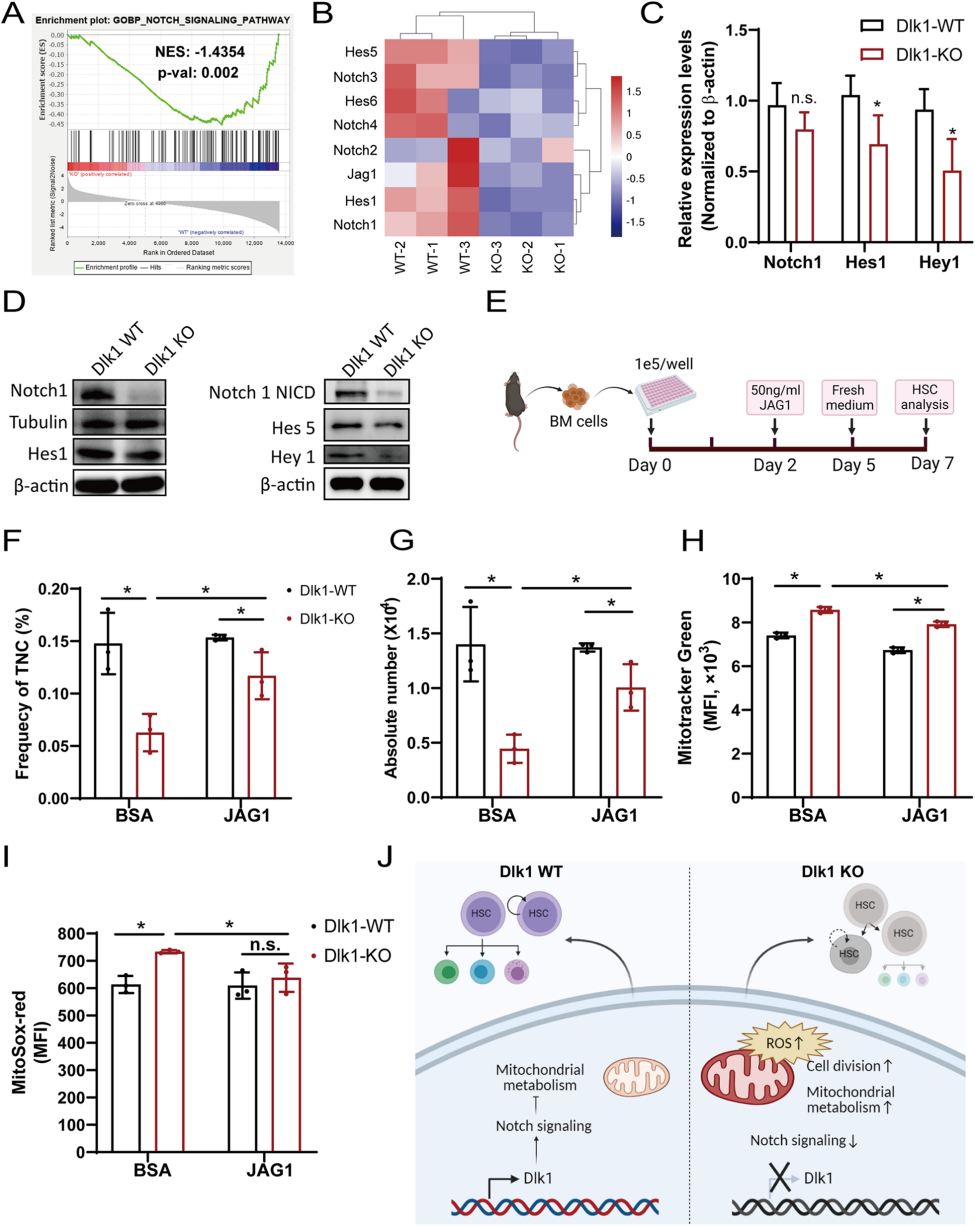
Dlk1 repressed mitochondrial metabolism through Notch signaling in adult mice HSCs. **A** GSEA analysis of the Notch signaling pathway in Dlk1 knockout LSK cells. **B** Heatmap of critical genes of Notch signaling pathway in LSK (Dlk1 WT versus Dlk1 KO). **C** qRT-PCR validating the gene expression of Notch1, Hes1 and Hey1. **D** Western blot validating the protein expression of Notch1, Hes1, Notch1 NICD, Notch3, Hey1 and Hes5. **E** Schematic diagram of Notch pathway agonist JAG1 incubation with bone marrow cells during in vitro culture. The SLAM HSCs (LSK, CD150^+^ and CD48^−^) were gated for analysis in **F**–**I**. **F**, **G** Frequency in TNC and absolute number of SLAM HSCs per legs. **H** Analysis of mitochondrial mass by Mitotracker green in HSCs (n = 3). **I** Analysis of mitochondrial superoxide by MitoSOX Red in HSCs (n = 3). **K** Model of this study. Data were expressed as mean ± SD; *p < 0.05; **p < 0.01; ***p < 0.001

As mentioned above, Dlk1 has been regarded as a non-canonical Notch ligand, and previous studies suggested that Dlk1 could exert its activity through activation or inhibition of Notch signaling under different cellular contexts [8, 9]. Notch signaling has been reported to regulate mitochondrial metabolism in breast cancer cells and in proinflammatory macrophage [34, 35] and was essential in embryonic hematopoiesis process [36]. Thus, we wondered whether Dlk1 knockout-induced phenotypes in HSCs were mediated by Notch signaling. To this end, we thoroughly analyzed the changes in Notch signaling pathway in RNA-Seq data and found that it was notably inhibited by Dlk1 knockout (Fig. 6A). Some critical genes in Notch signaling pathway was distinctly downregulated (Fig. 6B). We further experimentally examined the expression of some of these genes, and we showed that the mRNA levels of Notch1, Hes1 and Hey1 and protein expression of Notch1, active form of Notch1 (NICD), Notch3, and Notch downstream genes Hes1, Hey1, Hes5 were distinctly decreased after Dlk1 knockout in HSCs (Fig. 6C, D, Additional file 9: Fig. S9A). To further verify the involvement of Notch signaling in Dlk1 knockout-induced changes in HSCs, we re-activated the Notch signaling with an exogenous Notch ligand JAG1 in the context of Dlk1 knockout in HSCs (Fig. 6E). We firstly confirmed that exogenous JAG1 could partly reverse the inhibitory effect on Notch downstream gene expression (Hes1, Hey1 and Hes6) by Dlk1 KO (Additional file 9: Fig. S9B–D), indicating that JAG1 indeed activated Notch signaling. We then found that re-activation of Notch signaling could largely rescue the alterations in frequency and absolute number of HSCs (Fig. 6F, G), and reverse the over-activated mitochondrial metabolic activities of HSCs induced by Dlk1 knockout (Fig. 6H, I). We further over-expressed (OE) Notch1 active form NICD in the Dlk1 WT and Dlk1 KO HSCs to activate the Notch signaling (Additional file 9: Fig. S9E) and examined the alterations in HSC frequency and number and mitochondrial ROS levels. We found that activating Notch signaling under Dlk1 knockout or not by NICD overexpression had similar rescue effects on HSC frequency and number and mitochondrial ROS level with adding exogenous Notch ligand JAG1 (Additional file 9: Fig. S9F–H). Overall, these data showed that in adult mice HSCs, Dlk1 knockout could expand phenotypic HSCs, but virtually undermine their hematopoietic repopulation potential, which was partially due to inhibition of Notch signaling leading to over-activation of mitochondrial metabolism (Fig. 6J, Additional file 10: Table S1).

## Discussion

Dlk1-Gtl2 imprinted gene cluster has been demonstrated to regulate the function of mice fetal liver LT-HSCs, which involved epigenetic regulation and mitochondrial function and energy metabolism [2]. Dlk1 itself expressed in mice stromal cells of aorta-gonad-mesonephros hematopoietic microenvironment could negatively regulate the emergence of HSPC during embryonic period [17]. Previously, by global transcription profiling of 17 hematopoietic cell types of mice, we unexpectedly found that Dlk1 was highly expressed in hematopoietic stem and progenitor cells of mice. Noteworthy, Dlk1 presented the highest expression in LT-HSCs. In this study, we specially analyzed the Dlk1 expression levels by online hematological databases and did flow cytometry and qRT-PCR analysis. We confirmed that Dlk1 indeed had a unique expression pattern in adult mice HSCs (Fig. 1A–C, Additional file 1: Fig. S1A–C). This made us wonder whether Dlk1 has a unique function in adult mice HSCs.

We specifically knockout Dlk1 in adult mice HSCs and examined the changes in bone marrow by series of flow cytometric analysis. We observed that Dlk1 knockout exhibited a significant expansion of phenotypically defined LT-HSCs and entry of dormant HSCs into cell cycle (Fig. 1F–H). Studies have demonstrated that cell cycle regulation in HSCs is controlled exquisitely, which could be affected by extrinsic cues and intrinsic regulatory pathways. Deregulation of cell cycle could lead to transformation of hematopoietic stem and progenitor cells into leukemia-initiating stem cells, or lead to excessive proliferation and depletion of HSCs [37]. Dlk1 knockout-induced expansion of LT-HSCs and cell cycle activation suggested that Dlk1 as a transmembrane ligand could impact regulatory pathways related to cell cycle, which further influence the dormant state and self-renewal of HSCs. Previous study has suggested that conventional Dlk1 knockout (Dlk1-/-) in mice could change the numbers of different types of B cells in spleen and bone marrow, indicating that Dlk1 essentially regulate normal B cell development [10]. In our conditional knockout mice model (Mx1-cre), we showed that Dlk1 knockout could also decrease the frequency and absolute number of mature B cells, although with no significance (Fig. 1I–L). This suggested that Dlk1 could regulate differentiation and maturation of B cells. Besides, we also observed that Dlk1 knockout inhibited the erythrocyte, hinting that Dlk1 possibly could affect the erythrocyte development.

By competitive bone marrow transplantation assays, we demonstrated that the expanded LT-HSCs in Dlk1 knockout mice showed a defective long-term repopulating ability (Figs. 2B, L, 3B), suggesting that Dlk1 played a protective role in LT-HSCs function. Furthermore, we showed that Dlk1 knockout-induced phenotypes of LT-HSCs were due to the intrinsic changes in Dlk1 knockout LT-HSCs (Additional file 7: Fig. S7). However, previous study reported that Dlk1 from the stromal cells of hematopoietic microenvironment could act as negative regulator of HSPC emergence during embryonic hematopoiesis [17]. Dlk1-expressing fetal hepatic progenitors acting as supportive cells in coculture system could also support long term expansion of HSCs [38]. This suggested that Dlk1 acted differently in bone marrow microenvironment cells or supportive cells than in HSCs.

HSCs possess the ability to maintain its dynamic equilibrium among quiescence, proliferation, differentiation and aging. Many intracellular and extracellular factors may break this homeostasis and change the state of HSCs, in which metabolism dysfunction is a pivotal factor [1]. Generally, in embryonic hematopoiesis, high level of cell metabolic activities tends to couple with large amounts of nutrients and oxygen supply, which is required to meet the demand of continuous division and translocation of embryonic HSCs [39]. In this stage, increased metabolism level is beneficial for HSCs proliferation without disturbing the stemness of HSCs. However, in adult hematopoiesis, a majority of HSCs keep in a quiescence state and display low metabolic activity by maintaining low protein synthesis, low oxidation phosphorylation, and low ROS, in order to preserve HSCs pool and prevent excessive cell division [5, 28, 33]. At this point, an accident increase in metabolic activity of HSCs is regarded as a negative factor. For example, aging HSCs usually present high oxidative phosphorylation and high ROS level [40]. In this study, we demonstrated that Dlk1 knockout in HSCs could lead to entry of quiescent HSCs into the active cell cycle (Figs. 1H, 4C). It has been known that cell cycle regulation is critically important during hematopoiesis, and cell cycle tends to become more frequent when HSCs are differentiated into progenitor cells [37]. The proteins governing cell cycle, such as cyclin-dependent kinases could also regulate the HSCs self-renewal [41]. Thus, we thought that Dlk1 might function as a checkpoint protein of cell cycle in adult HSCs.

In the meantime, we found that Dlk1 knockout could increase ribosomal translation, glucose uptake and mitochondrial activities in LT-HSCs (Fig. 4D–L), which might be a consequence of increased cell division of LT-HSCs. Increased mitochondrial activity and oxidative phosphorylation tends to result in ROS overproduction [42]. ROS-induced DNA damage can impair the self-renewal of HSCs. ROS can also activate proliferation, followed by differentiation, exhaustion or apoptosis of HSCs [43]. Therefore, we proposed that Dlk1 knockout could activate the cell division of adult mice LT-HSCs, and lead to increased mitochondrial activity and ROS overproduction that could be an essential reason of impairment of hematopoietic repopulation potential.

Several in vitro studies suggested that Dlk1 was a non-canonical ligand of Notch signaling, and it could inhibit the Notch signaling [44–46]. However, in our in vivo results, we showed that in adult mice HSCs, Dlk1 could positively regulate the Notch signaling (Fig. 6A–D), since Dlk1 knockout in HSCs attenuated the Notch signaling. We further showed that reactivation of Notch signaling by exogenous ligand JAG1 under Dlk1 knockout in HSCs could largely rescue the alterations in absolute number and frequency of HSCs, and inhibit the mitochondrial activity and ROS level (Fig. 6F–I). Another intriguing question was that how JAG1 could rescue Notch signaling when Dlk1 knockout reduced both the reduce the levels of both receptor (Notch1/3/4) and ligand Jag1, we thought it could be attributed to that although Dlk1 knockout reduced levels of both receptor and ligand Jag1, yet it did not completely abrogate their expression or inhibit activity of Notch receptors. The exogenous Jag1 in the medium still could activate (at least partially activated) Notch signaling. Previous studies have suggested that Notch receptor or ligand could regulate cell cycle by maintaining HSCs quiescence [47], or change cell cycle kinetics to affect hematopoietic progenitor cell differentiation. Activation of Notch signaling could inhibit proliferation and survival of human hematopoietic progenitor cells [48]. Notch signaling could also induce cell cycle arrest in myeloid leukemia [49]. These studies indicated that in adult mice HSCs, Dlk1 functioned as a positive regulator of Notch signaling that mainly governed the state of cell cycle.

## Conclusion

In summary, we uncovered that in adult mice HSCs, Dlk1 played a protective role in maintaining the HSCs quiescence and homeostasis, which was depend on restricting the cell division and mitochondrial metabolic activity.

## Supplementary Information

**Additional file 1: Fig. S1.** Expression of Dlk1 in hematopoietic cells. (A-B) Dlk1 expression in blood cells. The data were analyzed in BloodSpot and Haemosphere database. (C) Schematic representation of the construction of the Dlk1 knockout mice and PCR genotyping in mouse offspring.

**Additional file 2: Fig. S2.** (A-C) The flow cytometry pattern of Fig. 1 F, I , K.

**Additional file 3: Fig. S3.** Dlk1 deletion did not render malignant hematopoiesis. (A) Representative photos of spleens from primary Dlk1 wild type and knockout mice. (B-C) Weight and absolute cell number of spleens of primary Dlk1 wild type and knockout mice. (D-I) Frequency in TNC and absolute number of HSPC (D and E), progenitor (F and G) and linage cells (H and I) in primary Dlk1 wild type and knockout mice (n = 6). (J) The hemogram analysis using the peripheral blood of Dlk1 wild type and knockout mice. Data were expressed as mean ± SD; *p < 0.05; **p < 0.01; ***p < 0.001. WT, wild-type mice. KO, knockout mice.

**Additional file 4: Fig. S4.** (A-D) The flow cytometry pattern of Figs. 1N, 2E, G, I.

**Additional file 5: Fig. S5.** (A-C) The flow cytometry pattern of Fig. 3E, G, I.

**Additional file 6: Fig. S6.** (A-C) The flow cytometry pattern of Fig. 3K, M, O.

**Additional file 7: Fig. S7.** The defective HSCs phenotype was not due to loss of Dlk1 in BM niche. (A-B) PB from 1st recipients were analyzed for percent donor repopulation at the indicated number of weeks after transplants and for percent mature donor-derived lineage cells at 16-week posttransplant. (C) The absolute number of donor-derived BM cells in 1st recipients were analyzed (n = 5). (D) Percent donor repopulation of 1st recipients at 16-week posttransplant (n = 5). (E–F) Frequency in TNCs and absolute numbers of HSPCs in 1st recipients at 16-week posttransplant (n = 5). Data were expressed as mean ± SD; *p < 0.05; n.s., no significance.

**Additional file 8: Fig. S8.** (A) Schematic diagram of the RNA-seq in this study. (B) Analysis of mitochondrial membrane potential by Dilc5 in Dlk1 wild type and knockout adult mice HSPC. (C) GSEA analysis of the pathways related to cellular translation and mitochondrial activity.

**Additional file 9: Fig. S9.** (A) Protein expression of Notch3 in Dlk1 WT and KO HSPC of mice. (B-D) Gene expression of Notch downstream genes Hes1, Hey1 and Hes6 in Dlk1 WT and KO HSPC after co-culture with Notch1 ligand JAG1. (E) Western blot result validating that Notch1 active form NICD was overexpressed in Dlk1 WT and KO HSPC of mice after lentivirus infection. (F–H) The changes in frequency and absolute number of SLAM HSCs (LSK, CD150^+^, CD48^−^) after NICD overexpression in Dlk1 WT and KO HSPC of mice (n = 3). (G) Analysis of mitochondrial ROS in Dlk1 WT and KO HSPC after NICD overexpression (n = 3). Data were expressed as mean ± SD; *p < 0.05; **p < 0.01; ***p < 0.001.

**Additional file 10: Table S1.** Primers used in this study.

## Data Availability

The data underlying this article will be shared on reasonable request to the corresponding author.
