# Supplementary figures and images for: Dlk1 maintains adult mice long-term HSCs by activating Notch signaling to restrict mitochondrial metabolism
Source: Exp Hematol Oncol. 2023 Jan 18;12:11. doi: 10.1186/s40164-022-00369-9 (PMC9850540; doi:10.1186/s40164-022-00369-9)

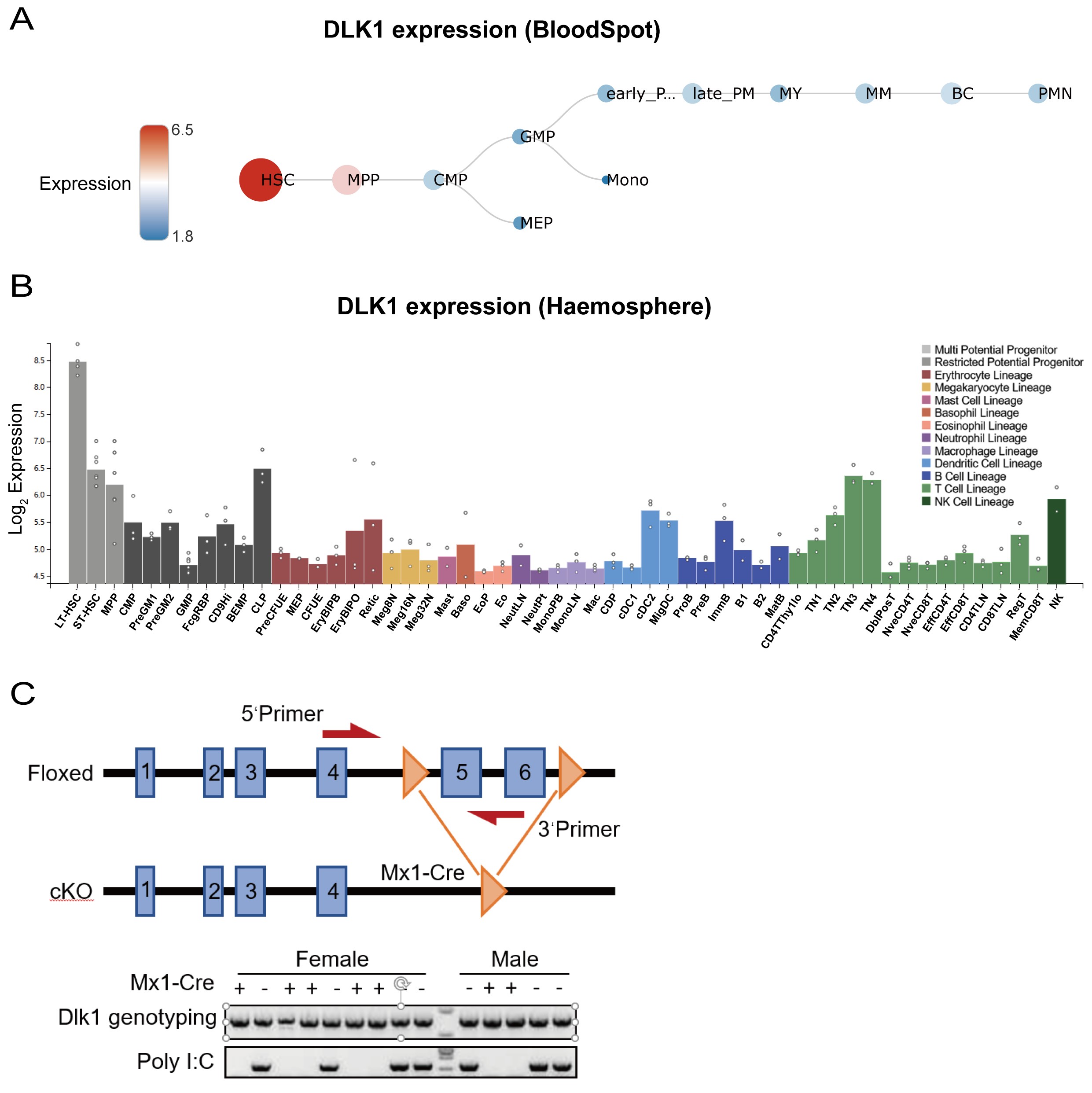

Supplement: Supplementary file 1 — Additional file 1: Fig. S1. Expression of Dlk1 in hematopoietic cells. (A-B) Dlk1 expression in blood cells. The data were analyzed in BloodSpot and Haemosphere database. (C) Schematic representation of the construction of the Dlk1 knockout mice and PCR genotyping in mouse offspring. [file 40164_2022_369_MOESM1_ESM.tif]

**DIk1 KO**

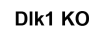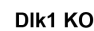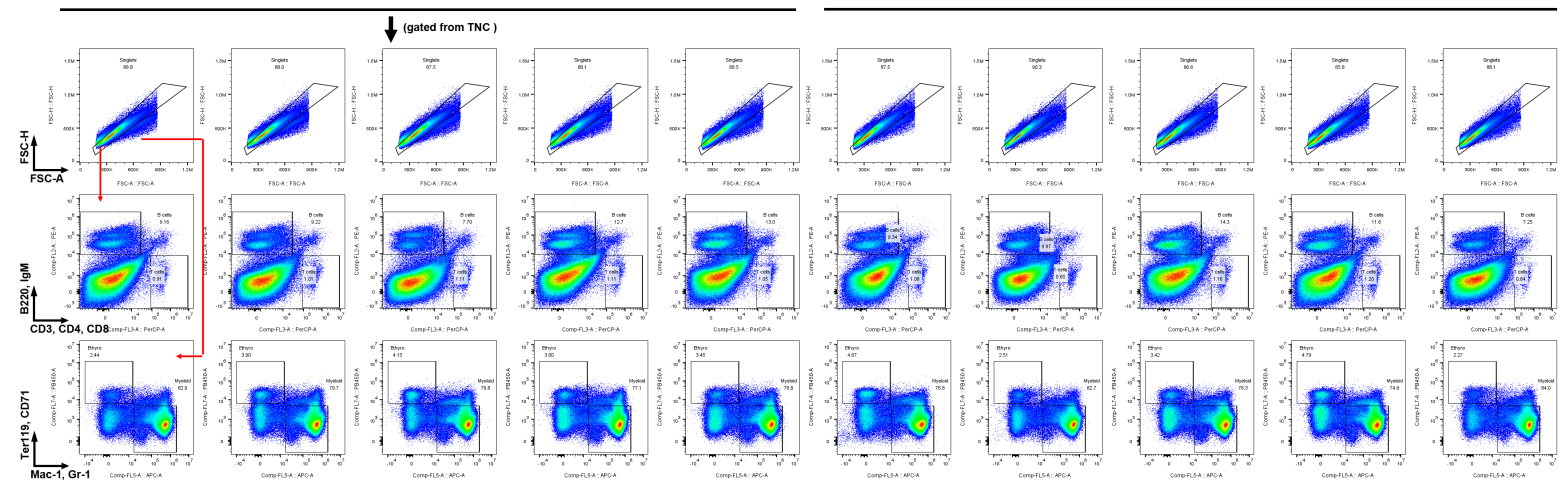

Supplement: Supplementary file 2 — Additional file 2: Fig. S2. (A-C) The flow cytometry pattern of Fig. 1 F, I , K. [file 40164_2022_369_MOESM2_ESM.pdf]

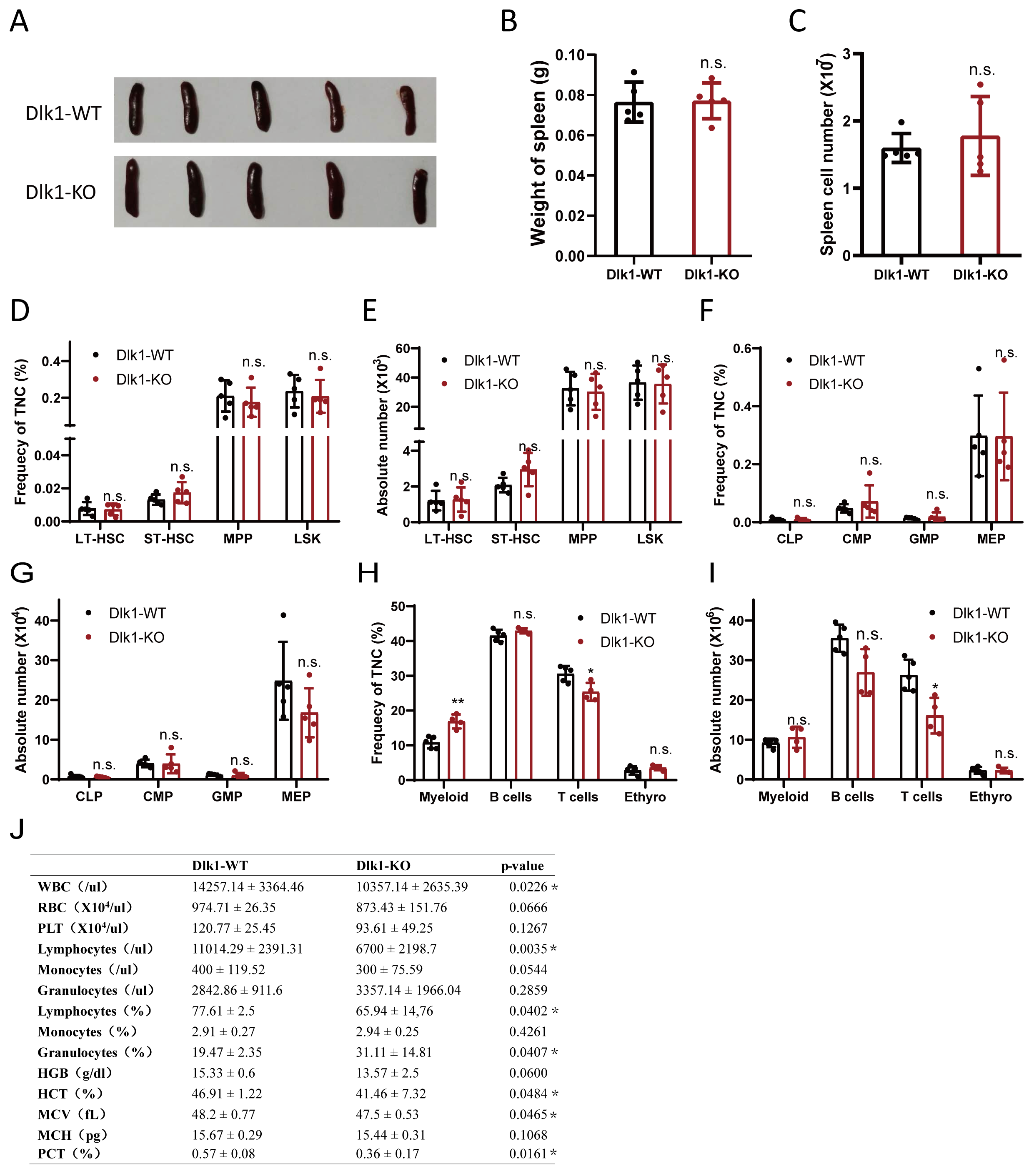

Supplement: Supplementary file 3 — Additional file 3: Fig. S3. Dlk1 deletion did not render malignant hematopoiesis. (A) Representative photos of spleens from primary Dlk1 wild type and knockout mice. (B-C) Weight and absolute cell number of spleens of primary Dlk1 wild type and knockout mice. (D-I) Frequency in TNC and absolute number of HSPC (D and E), progenitor (F and G) and linage cells (H and I) in primary Dlk1 wild type and knockout mice (n = 6). (J) The hemogram analysis using the peripheral blood of Dlk1 wild type and knockout mice. Data were expressed as mean ± SD; *p < 0.05; **p < 0.01; ***p < 0.001. WT, wild-type mice. KO, knockout mice. [file 40164_2022_369_MOESM3_ESM.tif]

A

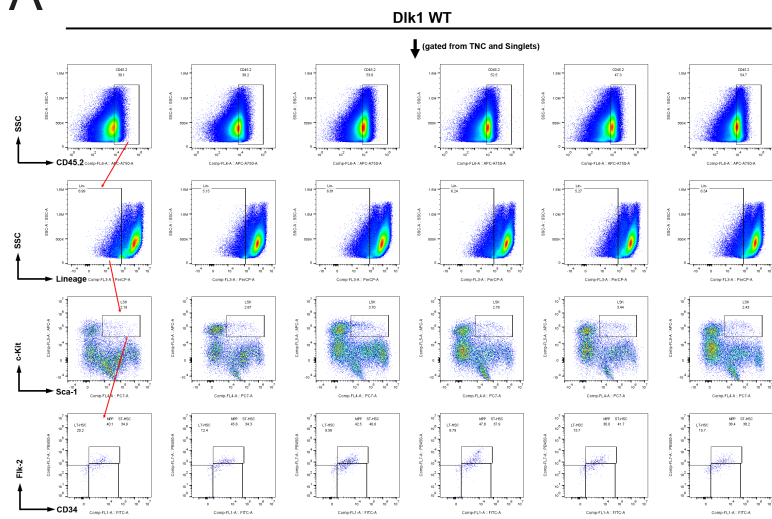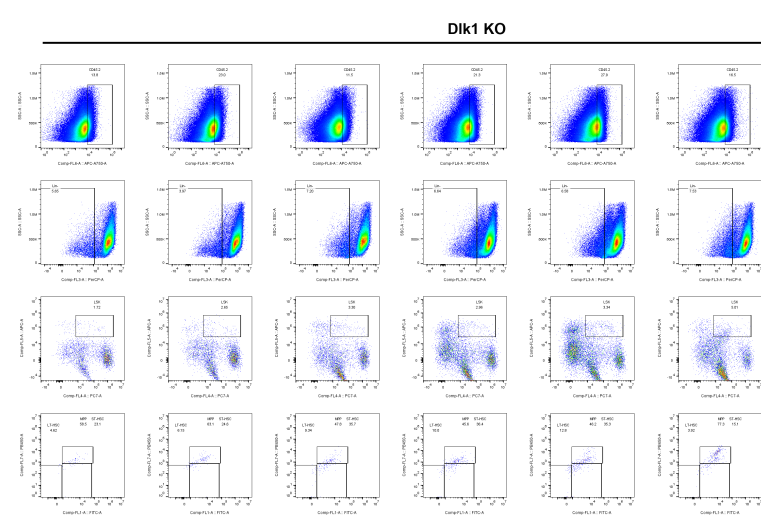

B

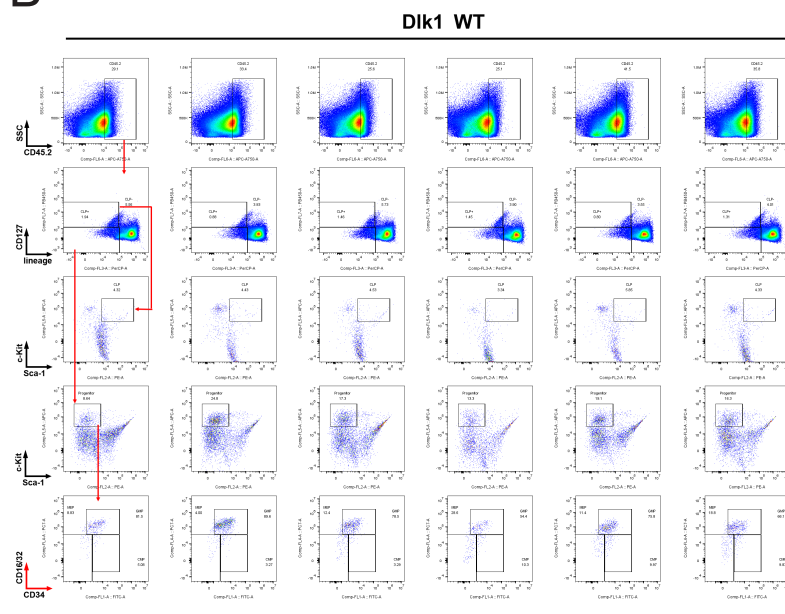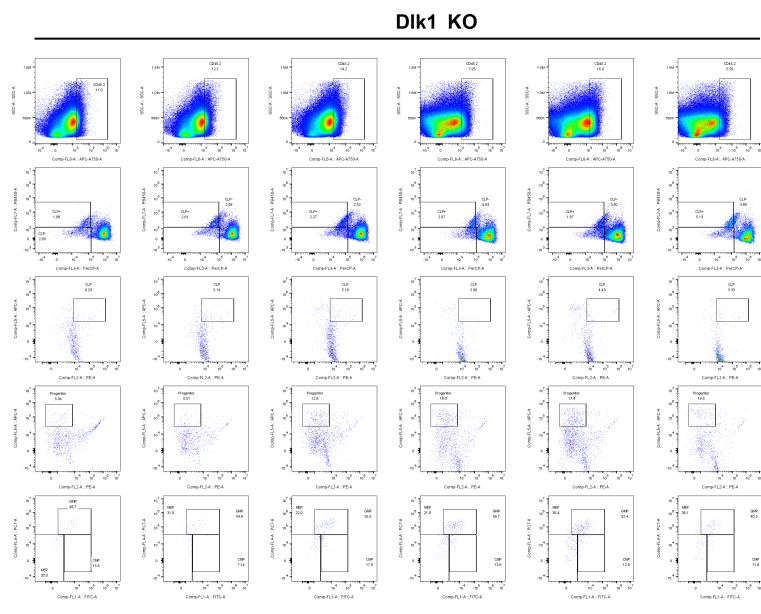

C

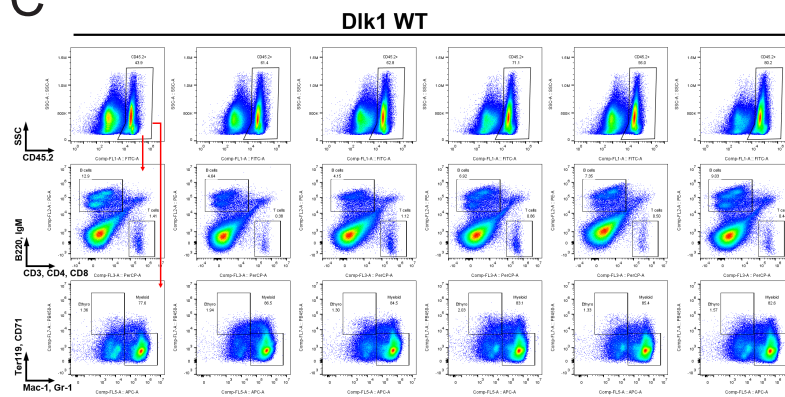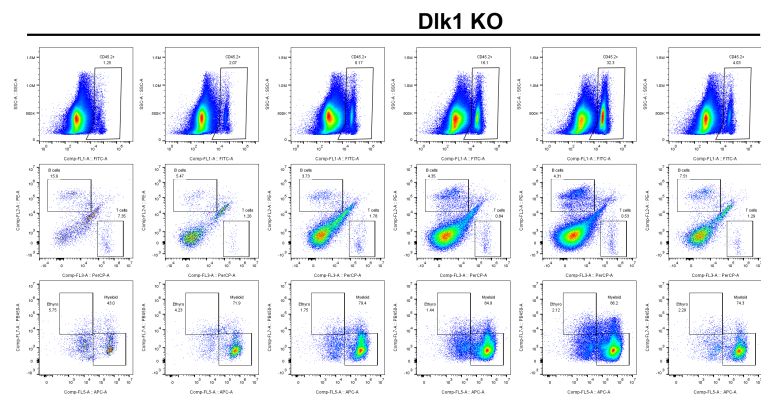

Supplement: Supplementary file 5 — Additional file 5: Fig. S5. (A-C) The flow cytometry pattern of Fig. 3E, G, I. [file 40164_2022_369_MOESM5_ESM.pdf]

### DIK1 KO

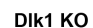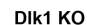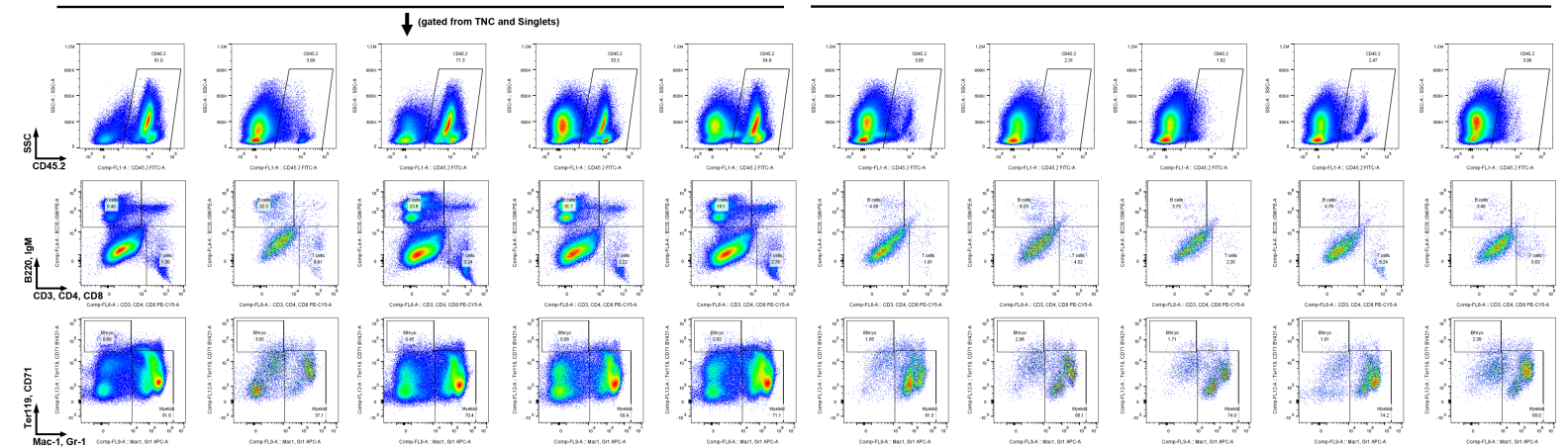

Supplement: Supplementary file 6 — Additional file 6: Fig. S6. (A-C) The flow cytometry pattern of Fig. 3K, M, O. [file 40164_2022_369_MOESM6_ESM.pdf]

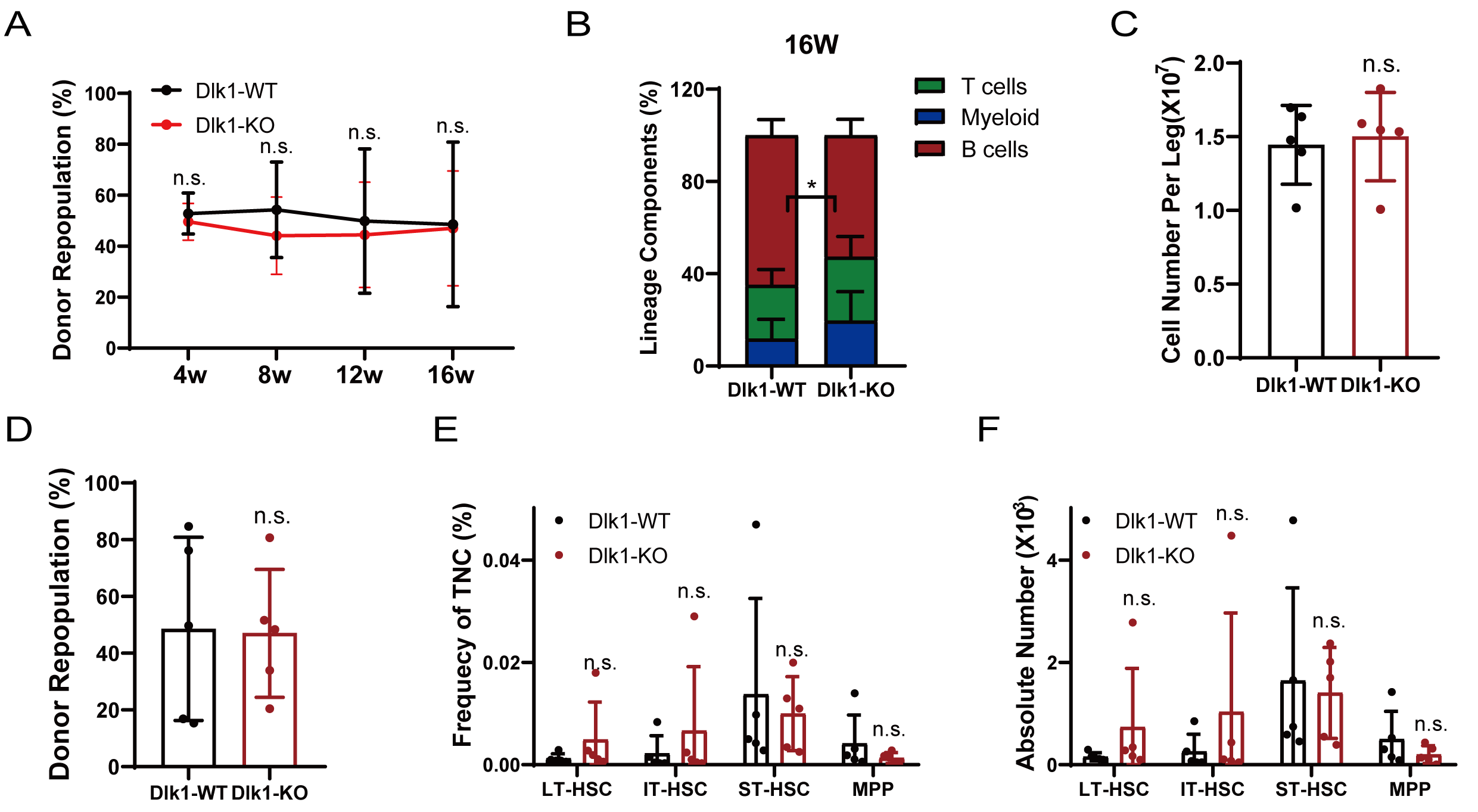

Supplement: Supplementary file 7 — Additional file 7: Fig. S7. The defective HSCs phenotype was not due to loss of Dlk1 in BM niche. (A-B) PB from 1st recipients were analyzed for percent donor repopulation at the indicated number of weeks after transplants and for percent mature donor-derived lineage cells at 16-week posttransplant. (C) The absolute number of donor-derived BM cells in 1st recipients were analyzed (n = 5). (D) Percent donor repopulation of 1st recipients at 16-week posttransplant (n = 5). (E–F) Frequency in TNCs and absolute numbers of HSPCs in 1st recipients at 16-week posttransplant (n = 5). Data were expressed as mean ± SD; *p < 0.05; n.s., no significance. [file 40164_2022_369_MOESM7_ESM.tif]

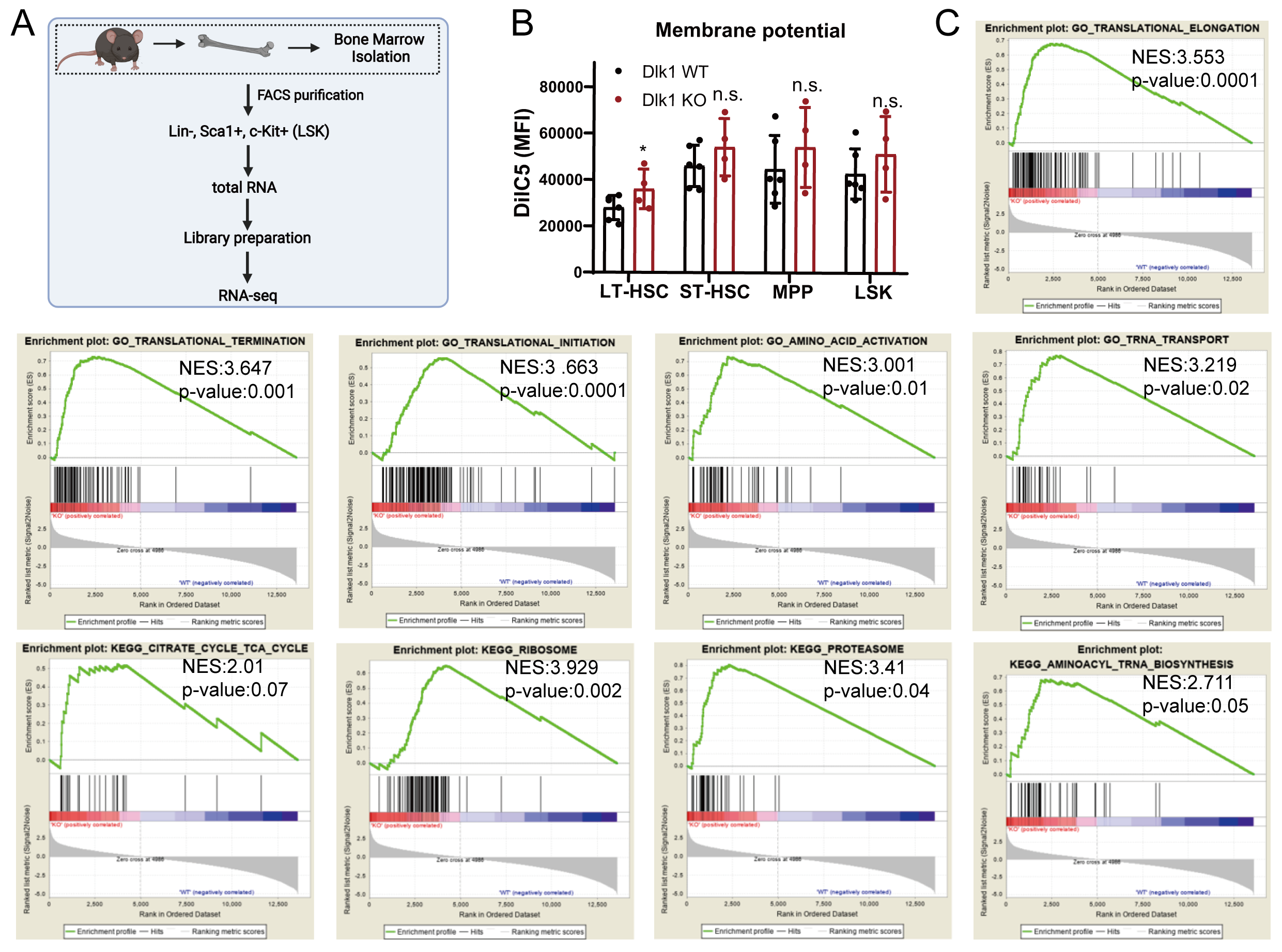

Supplement: Supplementary file 8 — Additional file 8: Fig. S8. (A) Schematic diagram of the RNA-seq in this study. (B) Analysis of mitochondrial membrane potential by Dilc5 in Dlk1 wild type and knockout adult mice HSPC. (C) GSEA analysis of the pathways related to cellular translation and mitochondrial activity. [file 40164_2022_369_MOESM8_ESM.tif]

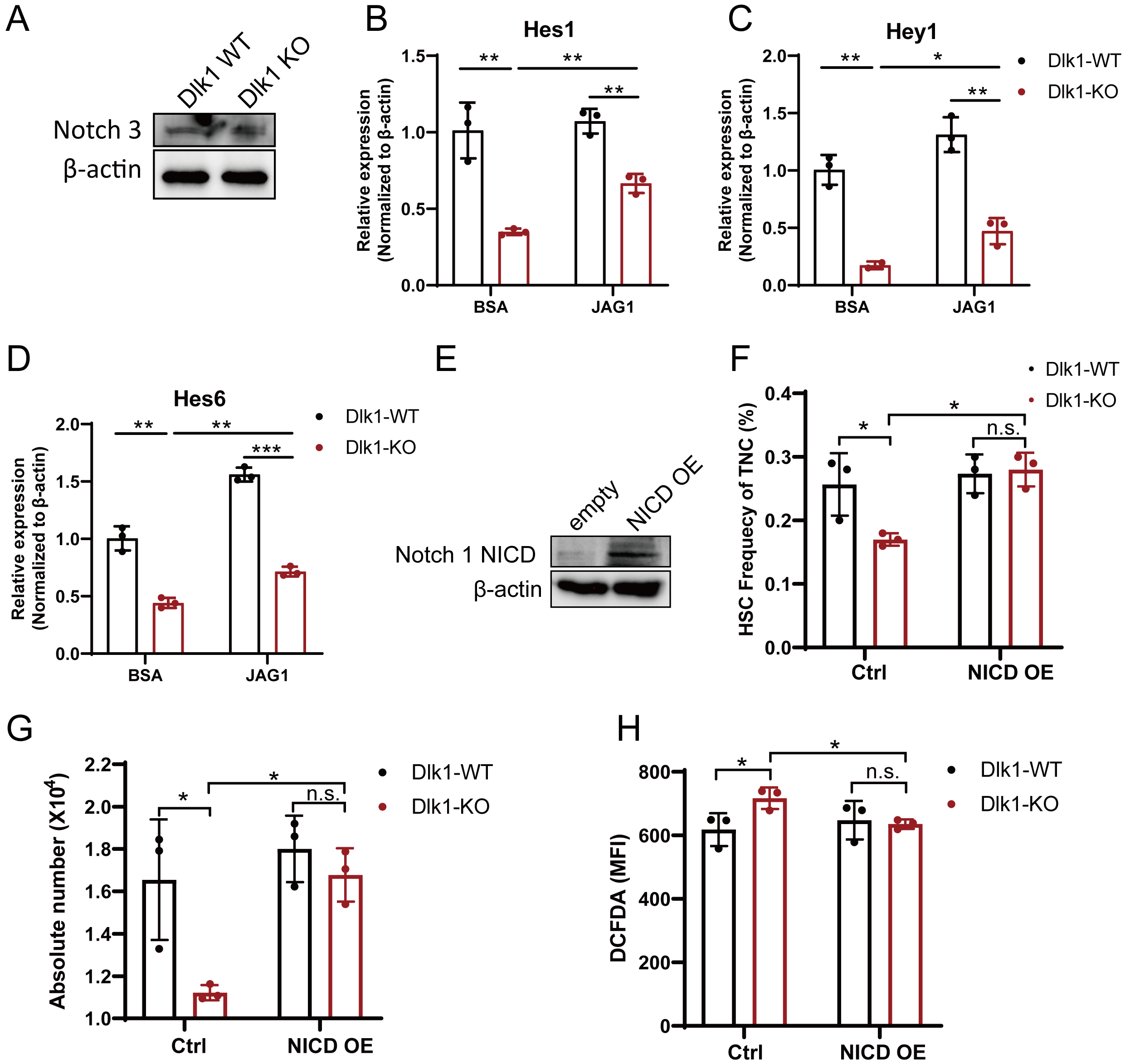

Supplement: Supplementary file 9 — Additional file 9: Fig. S9. (A) Protein expression of Notch3 in Dlk1 WT and KO HSPC of mice. (B-D) Gene expression of Notch downstream genes Hes1, Hey1 and Hes6 in Dlk1 WT and KO HSPC after co-culture with Notch1 ligand JAG1. (E) Western blot result validating that Notch1 active form NICD was overexpressed in Dlk1 WT and KO HSPC of mice after lentivirus infection. (F–H) The changes in frequency and absolute number of SLAM HSCs (LSK, CD150+, CD48−) after NICD overexpression in Dlk1 WT and KO HSPC of mice (n = 3). (G) Analysis of mitochondrial ROS in Dlk1 WT and KO HSPC after NICD overexpression (n = 3). Data were expressed as mean ± SD; *p < 0.05; **p < 0.01; ***p < 0.001. [file 40164_2022_369_MOESM9_ESM.tif]
