# Supplementary material for: Dlk1 maintains adult mice long-term HSCs by activating Notch signaling to restrict mitochondrial metabolism
Source: Exp Hematol Oncol. 2023 Jan 18;12:11. doi: 10.1186/s40164-022-00369-9 (PMC9850540; doi:10.1186/s40164-022-00369-9)
Supplement: Supplementary file 10 — Additional file 10: Table S1. Primers used in this study. [file 40164_2022_369_MOESM10_ESM.docx]

**Table S1**: primers used in this study.

| Genes |  | Sequence |
| --- | --- | --- |
| Mouse Actin | Forward | CGTCGACAACGGCTCCGGCATG |
|  | Reverse | GGGCCTCGTCACCCACATAGGAG |
| Mouse Mx1 Cre | Forward | CGGTTATTCAACTTGCACCA |
|  | Reverse | GTGAGT TTCGTTTCTGAG CTCC |
| Mouse Dlk1 genotype | Forward | AGATTCCCCCACCTCCAAC |
|  | Reverse | TTCCCAAACTGGACATGAGC |
| Mouse Hes1 | Forward | GTCAACACGACACCGGACAA |
|  | Reverse | GGAATGCCGGGAGCTATCTTT |
| Mouse Hey1 | Forward | CGAGACCATCGAGGTGGAAAA |
|  | Reverse | TCGATGATGCCTCTCCGTCT |
| Mouse Hes6 | Forward | CAGGCCAGGAGGATGAGGA |
|  | Reverse | AGACTCTCGTTGATCCGTGC |
| Mouse Notch1 | Forward | CCGGTTTGAGGAGCCAGTAG |
|  | Reverse | GGCAATCATGAGGGGTGTGA |
