# Supplementary material for: Dlk1 maintains adult mice long-term HSCs by activating Notch signaling to restrict mitochondrial metabolism
Source: Exp Hematol Oncol. 2023 Jan 18;12:11. doi: 10.1186/s40164-022-00369-9 (PMC9850540; doi:10.1186/s40164-022-00369-9)
Supplement: Supplementary file 4 — Additional file 4: Fig. S4. (A-D) The flow cytometry pattern of Figs. 1N, 2E, G, I. [file 40164_2022_369_MOESM4_ESM.pdf]

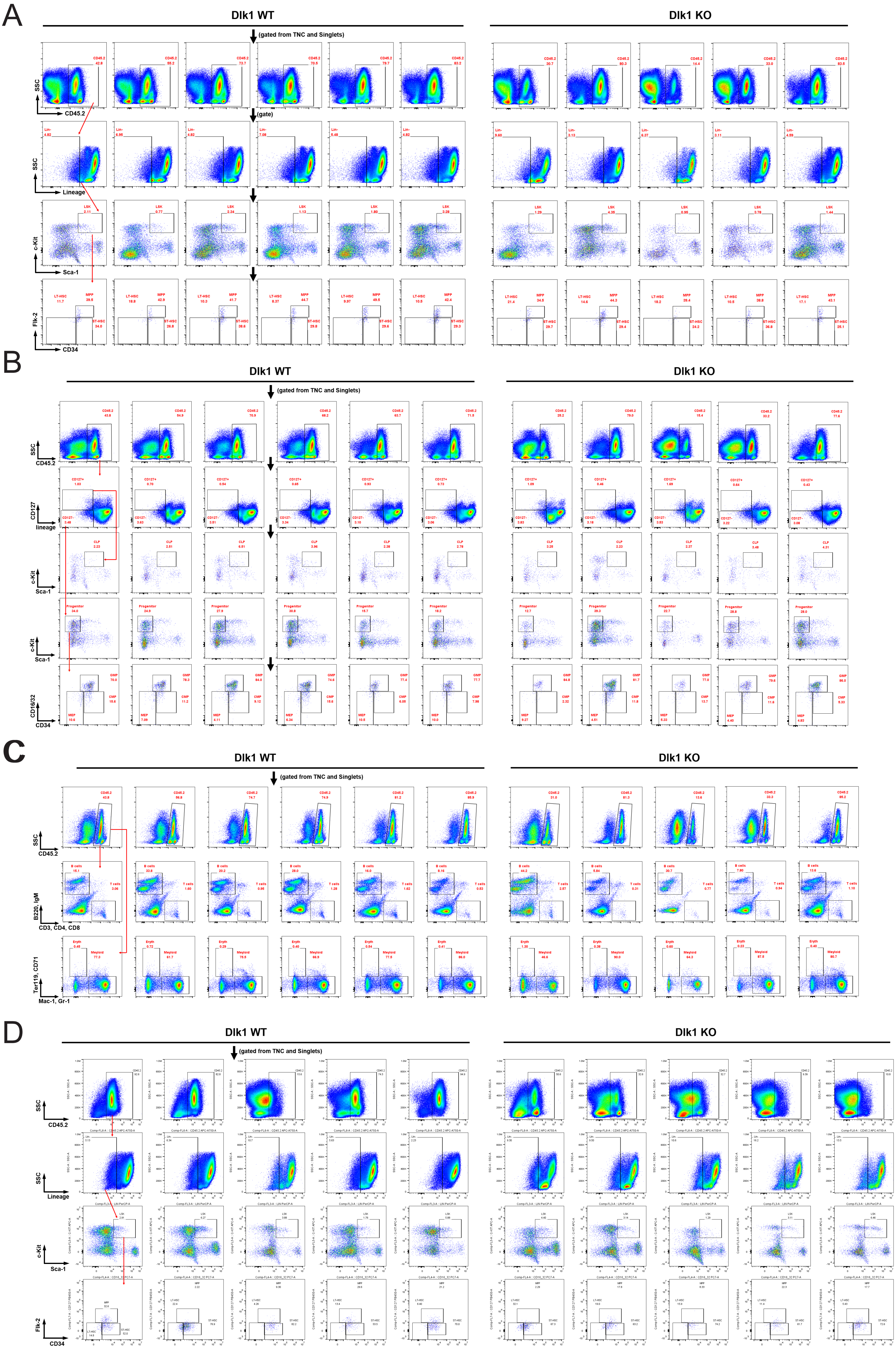
A
Dlk1 WT
Dlk1 KO
(gated from TNC and Singlets)
(gate)
SSC
CD45.2
Lineage
Lin-
c-Kit
Sca-1
LSK
Flk-2
CD34
LT-HSC
MPP
ST-HSC
B
Dlk1 WT
Dlk1 KO
(gated from TNC and Singlets)
SSC
CD45.2
CD127
lineage
CD127+
CD127-
c-Kit
Sca-1
CLP
Progenitor
CD16/32
CD34
GMP
CMP
MEP
C
Dlk1 WT
Dlk1 KO
(gated from TNC and Singlets)
SSC
CD45.2
B220, IgM
CD3, CD4, CD8
B cells
T cells
Ter119, CD71
Mac-1, Gr-1
Eryth
Meyloid
D
Dlk1 WT
Dlk1 KO
(gated from TNC and Singlets)
SSC
CD45.2
Lineage
c-Kit
Sca-1
Flk-2
CD34
